# Autophagy activity in cholangiocarcinoma is associated with anatomical localization of the tumor

**DOI:** 10.1371/journal.pone.0253065

**Published:** 2021-06-15

**Authors:** Gábor Lendvai, Tímea Szekerczés, Ildikó Illyés, Milán Csengeri, Krisztina Schlachter, Erzsébet Szabó, Gábor Lotz, András Kiss, Katalin Borka, Zsuzsa Schaff

**Affiliations:** 1 2nd Department of Pathology, Semmelweis University, Budapest, Hungary; 2 Department of Surgical and Molecular Pathology, Center of Tumor Pathology, National Institute of Oncology, Budpest, Hungary; Faculty of Medicine, University of Belgrade, SERBIA

## Abstract

The presence of autophagy has been indicated in cholangiocarcinoma (CC), which disease has poor prognosis and limited treatment options. Recently, CC has been classified by anatomical localization as intrahepatic (iCC), perihilar (pCC) and distal (dCC), showing different clinical and molecular characteristics. Thus, our aim was to compare autophagy activity in CC samples resected from different anatomical locations. Further, we investigated whether autophagy could be modulated in cell lines originated from iCC and extrahepatic CC (eCC) following the treatments with autophagy inhibitory and inducing agents. Tissue microarrays were prepared from 70 CC (28 iCC, 19 pCC and 23 dCC), 31 adjacent non-tumorous and 9 hepatocellular carcinoma (HCC) samples. Autophagy markers LC3, p62 and Beclin1 as well as proliferation marker Ki-67 were monitored by immunohistochemistry and were associated with patients’ survival. Modulation of autophagy was investigated in cell lines originated from iCC (HuH-28), eCC (TFK-1) and HCC (HepG2) by treating the cells with chloroquine (CQ) for inhibition and with Rapamycin, 5-Fluorouracil (5-FU) and Sorafenib for induction of autophagy. Our results indicated an inhibited autophagy in iCC and pCC tumor tissues, whereas active autophagy seemed to occur in dCC, especially in samples displaying low Ki-67 index. Additionally, low level of Beclin1 and high level of Ki-67 were associated with poor overall survival in dCC, suggesting the prognostic role of these proteins in dCC. Beside a baseline autophagy detected in each cell line, Rapamycin and 5-FU induced autophagy in iCC and HepG2 cell lines, Sorafenib in iCC cells. A chemotherapy agent in combination with CQ decreased IC50 effectively in the cell lines where basal and/or induced autophagy were present. In conclusion, we revealed differences in the autophagy activities of CC tissues and cell lines originated from different anatomical locations, which might influence patients’ treatment. Our results also suggest a prognostic role of Beclin1 and Ki-67 in dCC.

## Introduction

Cholangiocarcinoma (CC) is the second most common primary malignant liver tumor, which severely threatens human health with an increasing incidence rate, a generally poor prognosis and a five-year survival rate not reaching 30% [1]. Based on the different pathogenesis, clinical appearance and anatomical location, CC is classified as intrahepatic (iCC) and extrahepatic CC (eCC) and more recently as iCC, perihilar (pCC) and distal (dCC) forms [2–5].

Owing to the fact that CC is usually asymptomatic in early stages, most of the patients are at an advanced stage of the disease when diagnosed and treatment by surgical resection is limited [6]. As first-line treatment, cisplatin-based chemotherapy has been used for advanced CC [5,7]; nevertheless, the conventional therapeutic options do not lead to significant long-term survival and resistance to therapy occurs frequently. Further, cisplatin-based treatment implies severe toxicity, limiting the use of second-line palliative chemotherapy [8].

Autophagy is an intricate and conserved process involved in the elimination of damaged cellular organelles, including mitochondria and macromolecules, for recycling of bioenergy. The term autophagic flux refers to the sequestration of damaged organelles and unfolded proteins into autophagosomes and their degradation in autophago-lysosomes when autophagosomes fuse with lysosomes [9]. Autophagy has been studied in various diseases, for example in alcohol-induced, metabolic, and chronic viral hepatitis [10,11], and deciphering its role has contributed to better understanding of the pathogenesis of these diseases. During tumorigenesis, autophagy may act as a suppressor by reducing the damaged cellular components and proteins, thus helping to maintain homeostasis [12]. In autophagy-deficient cells, however, the accumulation of damaged mitochondria may lead to increased production of reactive oxygen species (ROS) and inhibited autophagy may be associated with over-activated proliferation pathways, promoting cell growth [13,14]. On the other side, however, an active basal and an adaptive stress-induced autophagy, being present in established tumors [11,14], may promote tumor growth by maintaining tumor cell survival under stressful conditions. Further, an active autophagy may even counteract anti-tumor treatments and contribute to the development of resistance [11,13,14].

In CC, risk factors and tumor-specific signaling pathways, such as inflammatory or proliferative pathways, are known to support or inhibit autophagy [13,15–17]. Since CC is a highly heterogeneous tumor [13,18], identification of molecular differences in CCs, classified as iCC, pCC and dCC, is needed, including autophagy activity. In line with this, we aimed to measure, compare, and correlate the expression of autophagy markers in tumorous and non-tumorous tissue samples of patients diagnosed with iCC, pCC, and dCC, and associate the levels of these markers with survival. Additionally, we investigated iCC- and eCC-derived cell lines for autophagy activity and also that whether different autophagy inhibitory and inducing agents, for example, second-line chemotherapies, would modulate autophagy activity in these cells.

## Materials and methods

### Patients’ characteristics and tissue samples

A total of 110 formalin-fixed paraffin embedded (FFPE) liver samples were collected from the archives of 2nd Department of Pathology at Semmelweis University, Budapest, including 28 cases of iCCs, 19 cases of pCCs, 23 cases of dCC, and 9 cases of HCC, as well as non-tumorous tissues adjacent to 10–10 cases of iCC (normal small bile ducts) and pCC (normal large bile ducts), and 11 cases of dCC (normal large bile ducts). The study comprised primary resected samples obtained between April 2004 and December 2017 from chemotherapy-naive patients. Descriptive parameters and histological characteristics of all cases are listed in Table 1. The patients were between 36 and 88 years of age and the male/female ratio was 29/41. The majority of the tumor cases were classified as Grade 2 (51.4% of CC, 66.7% of HCC) and Stage II (35.7% of iCC, 52.6% of pCC, 95.7% of dCC, and 44.4% of HCC). Overall survival (OS) was determined as the period of months lasted from the date of surgery until the date of patient’s death or the end of the follow-up. Permission for the retrospective analysis of the patients’ samples was obtained from the Committee of Scientific and Research Ethics, Budapest (45727-2/2013/EKU), without the inclusion of an informed consent, and the study was based on the ethical guidelines of the 1975 Declaration of Helsinki. The samples were fully anonymized before accessing them.

**Table 1:** The clinicopathological characteristics of patients diagnosed with intrahepatic and extrahepatic cholangiocarcinoma, and hepatocellular carcinoma.

	Intrahepatic CC	Extrahepatic CC		HCC
		pCC	dCC	
**Number of tumor samples**	28	19	23	9
**Number of surrounding non-tumorous samples**	10	10	11	-
**Number of cases by gender** (Male/Female)	7/21	10/9	12/11	8/1
**Average years of age**	60.5	64.3	66.0	64.2
**Median Overall Survival Time** (months)	20.67	7.27	14.9	ND
Grade^*^				
**I**	6	5	3	1
**II**	15	9	12	6
**III**	7	5	8	1
**IV**	0	0	0	1
Stage^*^^,^^**#**^				
	IA (6) IB (6) II (10) IIIB (1) IV (5)	I (1) II (10) IIIA (1) IIIB (1) IIIC (6)	IIA (6) IIB (16) IIIB (6)	IB (3) II (4) IIIA (1) IVB (1)

*Presented as number of patients

^#^Tumor-nodes-metastasis (TNM) classification was based on the 8^th^ edition.

CC: Cholangiocarcinoma, pCC: Perihilar cholangiocarcinoma, dCC: Distal cholangiocarcinoma, HCC: Hepatocellular carcinoma, ND: Not detected.

### Tissue microarray

Representatitve tumorous and non-tumorous areas of the hematoxylin-eosin (H&E) stained slides were selected by pathologists. From each cases, two core pieces of 2.0 mm in diameter were taken for the construction of tissue microarray (TMA) blocks. Cores from 79 tumors and 31 normal tissue donor blocks were placed in 5 TMA recipient blocks using an automated tissue-arraying instrument (3D HISTECH TMA Master II, Budapest, Hungary).

### Immunohistochemistry

Immunohistochemical (IHC) reactions were performed on 3–4 μm thick TMA sections using a Ventana Benchmark XT automatic immunostainer (Ventana Medical Systems; Tucson, AZ) with indirect streptavidin-biotin-peroxidase system. Following the antigen retrieval for 30 min at 95°C, the slides were incubated with the specific primary antibodies for 30 min at 42°C. The details of the used antibodies and positive controls are summarized in Table 2. The 3,3’-diaminobenzidine tetra-hydrochloride (DAB) substrate chromogen solution (iVIEW DAB Detection Kit, Ventana) was used for the visualization of IHC reactions followed by counterstaining with hematoxylin. The evaluation of the IHC reactions (except for Ki-67) was performed semi-quantitatively by determining the intensity of the staining (0 –no, 1 –minimal, 2 –weak, 3 –moderate, 4 –intensive and 5 –very intensive) and the percentage of positively stained cells: (0) < 6%, (1) 6–20%, (2) 21–40%, (3) 41–60%, (4) 61–80% and (5) >81%. The IHC score was defined as the sum of the intensity and percentage of positive reactions. For Ki-67, a percentage of positivity was determined according to the number of stained cells counting 100 tumor cells per a tissue core.

**Table 2:** The details of the antibodies used for immunohistochemistry and Western blot.

Primary antibody	Species	Manufacturer	Catalog Number	Dilution for IHC	Positive control for IHC	Dilution for Western blot
Beclin1, (BECN1, H-300)	polyclonal rabbit	Santa Cruz Biotechnology	sc-11427	1:100	cervical carcinoma/muscle	NA
LC3	polyclonal rabbit	Novus Biologicals	NB-100-2331	1:200	cervical carcinoma	NA
p62	monoclonal mouse	Abcam	ab56416	1:1000	hepatocellural carcinoma	1:1250
TOMM20 (F-10)	monoclonal mouse	Santa Cruz Biotechnology	sc-17764	1:200	cervical carcinoma	NA
COX4 (F-8)	monoclonal mouse	Santa Cruz Biotechnology	sc-376731	1:100	rectal carcinoma	NA
Ki-67 (AC3)	monoclonal mouse	Dako	274–11	1:200	tonsilla	NA
LC3	polyclonal rabbit	Cell Signaling	4108	NA	NA	1:1000
TOMM20 (FL-145)	polyclonal rabbit	Santa Cruz Biotechnology	sc-11415	NA	NA	1:200
β-actin (AC-15)	monoclonal mouse	Sigma-Aldrich	A5441	NA	NA	1:2000

NA: Not applicable.

### Cell lines and culturing

The human cell line HuH-28 (originated from iCC) [19]; TFK-1 (originated from eCC) [20] and HepG2 (originated from hepatoma) [21] were a kind gift of Stephanie Roessler (Institute of Pathology, University of Heidelberg, Germany). The cell lines were maintained in RPMI-1640 medium (Gibco of Thermo Fisher Scientific Inc., Waltham, MA) supplemented with 10% fetal bovine serum (Cat No: P40-37500, Pan Biotech, Aidenbach, Germany), 1% Penicillin-Streptomycin (Cat No: P0781, Sigma-Aldrich, St. Louis, MO) and 1% L-Glutamine (Cat No: P04-08100, Pan Biotech). All cells were cultured at 37°C in a humidified chamber with 5% CO_2_ supplement.

### Drugs and treatments

Rapamycin, 5-Fluorouracil (5-FU), supplied by Sigma-Aldrich (Cat No: R0395 and F6627, repsectively), and Sorafenib, provided by Santa Cruz Biotechnology (Cat No: CAS284461-73-0, Dallas, TX) were dissolved in DMSO (Cat No: D2650, Sigma-Aldrich). Chloroquine (CQ), provided by Sigma-Aldrich (Cat No: C6628), was dissolved in water.

Seeding was carried out using 12-, 6- or 96-well plates (fluorescent visualization, autophagy induction, cell viability) and the number of cells varied accordingly: 4.5×10^5^, 1×10^5^ and 5×10^3^ cells of HuH-28, 5×10^5^, 1×10^5^ and 8×10^3^ cells of TFK-1, 3×10^5^, 8×10^4^ and 5×10^3^ of HepG2 cells. Higher number of cells was applied for seeding TFK-1 to compensate for its slower division rate compared to HuH-28 and HepG2. The cells were grown on coverslips when detecting mitochondrial morphology or autophagic vacuoles with fluorescent staining. Each treatment was carried out 24 h following seeding. Untreated or vehicle (DMSO)-treated cells served as controls.

Autophagy inhibition was performed by treating the cells with 50 μM of CQ for 24 h as single treatment or in combination with Rapamycin. For autophagy induction, cells were treated with 0.2 μM of Rapamycin for 24 h and, in separate experiments, with 5-FU (from 10 to 400 μM) or Sorafenib (from 5 to 20 μM) for 48 and 72 h. The effect on autophagy induction was examined by detecting the expression of autophagy-realted proteins with Western blot.

For investigating cell viability, the cells were treated with increasing concentrations of 5-FU (from 10 to 400 μM) or Sorafenib (from 5 to 25 μM) for 48 and 72 h. In order to examine whether inhibition of autophagy would affect the viability of chemotherapy-treated cells, 5-FU and Sorafenib were administered in combination with 50 μM of CQ for 48 and 72 h. Cell viability was determined by a proliferation assay.

### Detection of mitochondrial morphology

Following the removal of treatment media and a wash with ice-cold PBS (Lonza, Verviers, Belgium), the cells were incubated with 150 nM Mitotracker Orange CMTMRos (Cat No: M7510; Life Technologies of Thermo Fisher Scientific Inc., Eugenes, OR), diluted in serum-free medium, for 45 minutes at 37°C. The cells were fixed with 4% formaldehyde solution for 10 min, washed with acetone for 5 minutes and stained with 100 nM Mitoview Green (Cat No: 70054, Biotium Inc.; Fremont, CA) for 30 minutes at 37°C. For visualization, the coverslips with the stained cells were mounted to microscope slides using a Mounting Medium containing DAPI (Vectashield, Vector Laboratories, Inc. Burlingame, CA). The images were captured with a Leica DM-RXA Fluorescence Microscope (Leica, Wetzlar, Germany).

### Detection of the autophagosomes

For visualising autophagosomes, Monodansyl-cadaverine (MDC, Cat. No:D4008, Sigma Aldrich) was used according to the manufacturer’s guidelines. Briefly, the cells were stained with 0.1 mM of MDC for 30 min at 37°C, washed with PBS, fixed with 4% formaldehyde for 10 min at RT. For visualization, the coverslips with the stained cells were transferred to microscope slides and mounted with Fluorescent Mounting Medium (Cat No: S3023; DAKO, Glostrup, Denmark). The images were captured with a Leica DM-RXA Fluorescence Microscope (Leica).

### Western blot analysis

Following treatment, the cells were harvested using M-PER Mammalian Protein Extraction Reagent (Thermo Fisher Scientific Inc., Rockford, IL) supplemented with protease inhibitor cocktail (Cat No: P8340; Sigma-Aldrich). Protein content was determined by using Pierce BCA Protein Assay (Cat No: 23227; Thermo Fisher Scientific Inc.). Equal amounts (35 μg) of proteins were separated on a 15% SDS-polyacrylamide gel (ingredients supplied by Bio-Rad Laboratories Ltd., Hercules, CA) for 25 min at 85 V and 1 h at 150 V. A wet transfer of proteins was performed to a nitrocellulose membrane (Bio-Rad Laboratories Ltd.) for 90 min at 95 V. Non-specific binding was minimized with soaking the membrane in 5% non-fat dry milk, 1 x TBST for 1 h. Incubation with the primary antibodies, summarized in Table 2, was performed overnight at 4°C, followed by incubation with the corresponding secondary antibodies (horseradish peroxidase goat anti-mouse, Cat No: 31430, 1:2000, and horseradish peroxidase goat anti-rabbit, Cat No: 32460, 1:4000, both supplied by Thermo Fisher Scientific Inc.) for 2 h at RT. The bands were visualized using a highly sensitive enhanced chemiluminescence method (Clarity Max ECL Western Blotting Substrates, Bio-Rad Laboratories Ltd.). Images were taken by using a G:BOX Chemi XR 5 image documentation system (Syngene, Cambridge, UK). Intensity of the bands was quantified using ImageJ Software 1.46r (Bethesda, MD) and the expression of the analyzed proteins was normalized to the level of β-actin.

### Cell proliferations assay

The Sulforhodamine B (SRB) colorimetric assay was used for cytotoxicity screening. Following treatments, the cells were fixed with 70 μl of 10% trichloroacetic acid (TCA) for 1 h at 4°C, rinsed 5 times with tap water, and air-dried. Next, the cells were stained with 70 μl of 0.4% SRB (S1402, Sigma-Aldrich), 1% acetic acid solution for 15 min. Following a repeated wash with 1% acetic acid and air-drying, the cell-associated stain was dissolved in 200 μl of 10 mM Tris-HCl (pH 8). The absorbance was determined at 570 nm by using an EL-800 microplate reader (BioTek Instruments, Bad Friedrichshall, Germany). The data were normalized to control (untreated) and expressed as cell viability (% of control).

### Statistical analysis

The analyses of IHC scores (tumorous versus non-tumorous areas and iCC versus eCC) were examined by a non-parametric Mann-Whitney U test. The group-wise statistical testing (iCC, pCC and dCC) was performed by a non-parametric Kruskal-Wallis analysis of variance. Correlation between IHC scores and clinical data was investigated using a non-parametric Spearman rank correlation test. For survival analysis, the cases were divided into high and low protein expression groups based on the median IHC score for each analyzed protein. The Kaplan-Meier method was used to plot the survival curves and differences between the two groups were defined by the log-rank test. Statistical analysis of western blot results (autophagy induction by Rapamycin, 5-FU, Sorafenib, and cytotoxicity measurement) was carried out using the unpaired Student’s t-test. For chemotherapy treatment, IC50 values were determined by nonlinear regression. The statistical analyses were performed using GraphPad Prism (version 5.01; San Diego, CA) and Statistica v.13 (Stat-Soft Inc.,Tulsa, OK) software. A *p* value of 0.05 was set as the threshold for statistical significance.

## Results

### Evaluation of immunohistochemical staining

**Fig 1:**
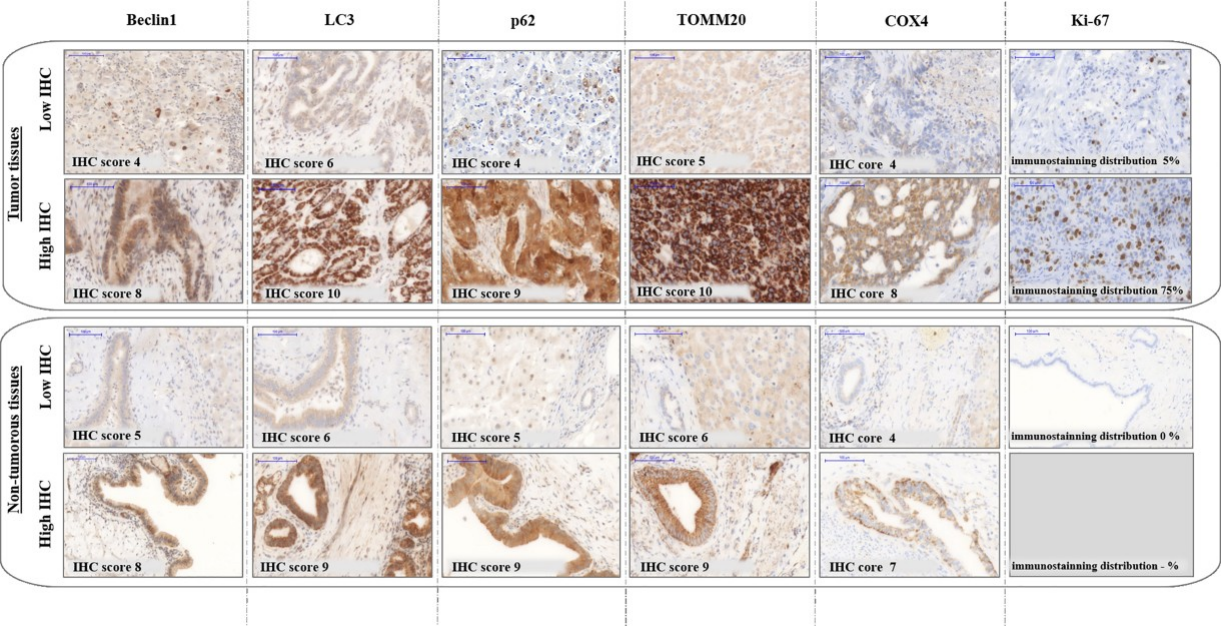
Representative low and high immunostainings. The images show Beclin1, LC3, p62, TOMM20, COX4 and Ki-67 expression detected in the resected tumorous and non-tumorous tissues. IHC: immunohistochemistry. The objective magnifications 20x.

We observed diffuse or occasionally dot-like LC3 and p62 staining with varying density, minimal Beclin1 and intensive TOMM20 reactions both in hepatocytes and cholangiocytes. Beclin1 displayed homogeneous expression in iCC, in HCC and partly in pCC; whereas the staining was mostly granular in dCC. For mitochondrial markers, TOMM20 revealed intensive granular cytoplasmic staining while COX4 was rather diffuse with a few granular reactions. The immunostaining of Ki-67 appeared to be nuclear and the expression was much higher in malignant tissues as compared to non-tumorous samples. Representative images of Beclin1, LC3, p62, TOMM20, COX4 and Ki-67 are shown in Fig 1.

**Fig 2:**
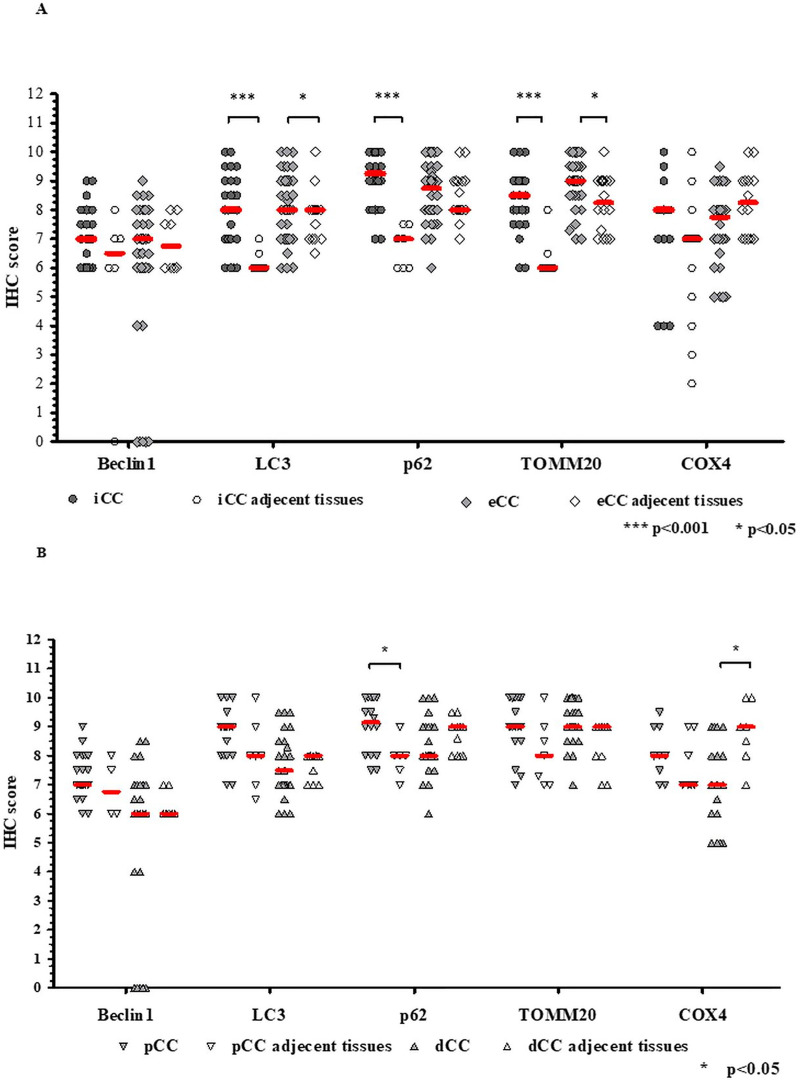
Expression of autophagy and mitochondria markers in cholangiocarcinoma (CC) subtypes in relation to surrounding areas detected by immunohistochemistry (IHC). (A) The levels of Beclin1, LC3, p62, TOMM20 and COX4 in iCC and eCC; (B) the levels of Beclin1, LC3, p62, TOMM20 and COX4 in pCC and dCC in comparison to adjacent non-tumorous surrounding areas. The thick horizontal lines represent the median. Statistical analysis was performed using a Mann-Whitney U test and the thin horizontal lines signify the statistical differences. Statistically significant differences are indicated by asterisks (*** *p* <0.001, ** *p* <0.01, * *p* <0.05). iCC: intrahepatic CC, eCC: extrahepatic CC, pCC: perihilar CC, dCC: distal CC.

When comparing iCC and eCC (pCC + dCC) with the adjacent non-tumorous surrounding tissues, increased LC3 (p<0.001), p62 (p <0.001) and TOMM20 (p <0.001) were found in iCC, elevated LC3 (p <0.05) and TOMM20 (p <0.05) were observed in eCC as compared to the surrounding tissues (Fig 2A). Further, the levels of LC3 were not different in pCC and dCC but p62 was increased in pCC (p <0.05) and COX4 was decreased in dCC (p <0.05) as compared to the adjacent tissues (Fig 2B). Beclin1 was not differently expressed between the tumorous and non-tumorous surrounding areas.

**Fig 3:**
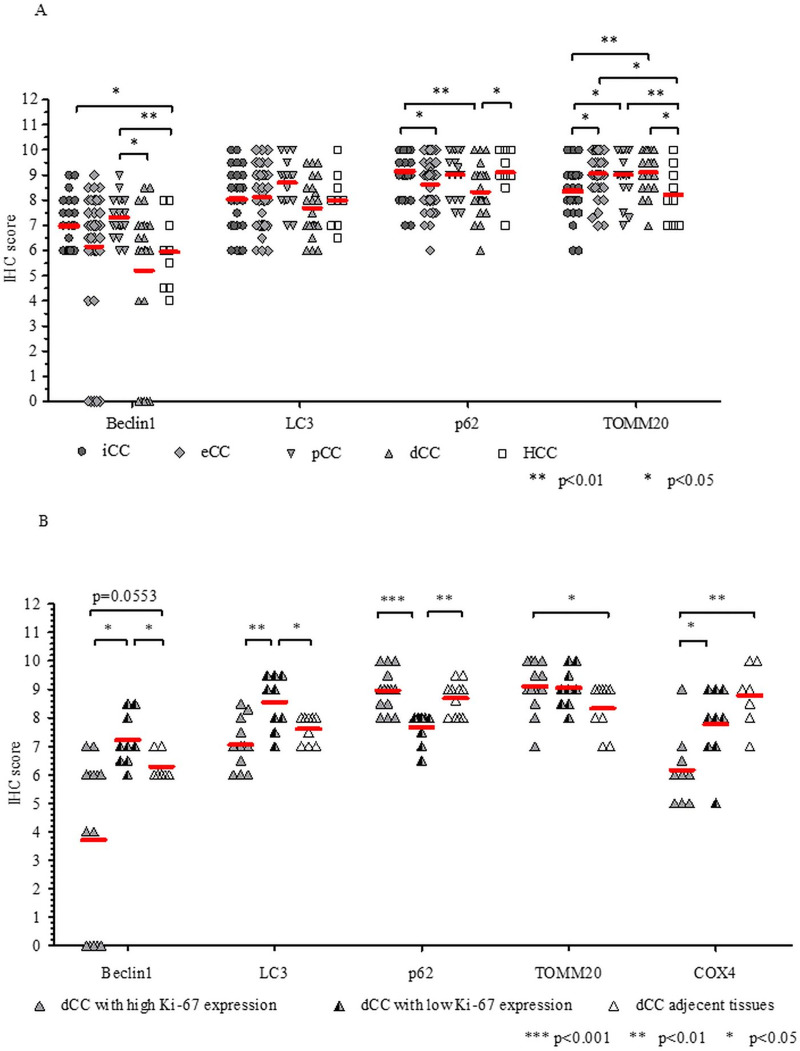
Expression of autophagy and mitochondria markers in cholangiocarcinoma (CC) subtypes and hepatocellular carcinoma (HCC) detected by immunohistochemistry (IHC). (A) The levels of Beclin1, LC3, p62 and TOMM20 in CC subtypes and HCC; (B) the levels of Beclin1, LC3, p62, TOMM20 and COX4 in low and high Ki-67-expression subgroups of dCC and in non-tumorous tissues adjacent to dCC. The thick horizontal lines represent the median. Statistical analysis was carried out by using Kruskal-Wallis analysis of variance (A) and a Mann-Whitney U test (B). The thin horizontal lines signify the statistical differences. Statistically significant differences are indicated by asterisks (*** *p* <0.001, ** *p* <0.01, * *p* <0.05). iCC: intrahepatic CC, eCC: extrahepatic CC, pCC: perihilar CC, dCC: distal CC.

For comparing iCC and eCC, reduced level of p62 (p <0.05) and increased expression of TOMM20 (p <0.05) were observed in eCC as compared to iCC (Fig 3A). Regarding pCC and dCC, p62 was found to be decreased in dCC (*p* <0.01) and TOMM20 was increased in both pCC (*p* <0.05) and dCC (*p* <0.01) when compared to iCC; whereas Beclin1 was decreased in dCC as compared to pCC (*p* <0.05). In relation to HCC, increased Beclin1 was found in both iCC (*p* <0.05) and pCC (*p* <0.01), reduced p62 was observed in dCC (*p* <0.05), and increased TOMM20 was detected in eCC, and also in pCC and dCC (p<0.05). The IHC scores of LC3 and COX4 (S1 Fig) showed no differences between the CC groups.

The Ki-67 index was found to be low in iCC, pCC and HCC (15% for iCC and 25% for pCC and HCC), however, it was remarkably different in dCC (70%, ranging from 20 to 90%). As Ki-67 expression was not homogenous across dCC samples, we divided the cases into high- and low-Ki-67 expression subgroups based on the median value and reperformed the statistical analysis (Fig 3B). Comparing to adjecent non-tumorous tissues, the levels of Beclin1 (*p* <0.05) and LC3 (*p* <0.05) were increased and the expression of p62 (*p* <0.01) was decreased in the low Ki-67-expression subgroup; whereas the level of TOMM20 was increased (*p* <0.05) and that of COX4 was decreased (*p* <0.01) in the high Ki-67-expression subgroup. The decreased Beclin1 observed in the high Ki-67-expression subgroup in comparison to the adjacent tissues did not reach the set threshold for statistical significance (p = 0.0553). Comparing the low and high Ki-67 subgroups, the levels of Beclin1 (*p* <0.05), LC3 (*p* <0.01), and COX4 (*p* <0.05) were increased and the expression of p62 (*p* <0.001) was decreased in low Ki-67-expression subgroup as compared to the high Ki-67 subgroup. When considering these expression levels in relation to iCC and pCC (Fig 3A and 3B, statistics is not shown), we found decreased Beclin1 in the high Ki-67-expression subgroup as compared to iCC and pCC (*p* <0.01) and decreased LC3 as compared to pCC (*p* <0.01). Further, we found decreased p62 in the low Ki-67-expression subgroup as compared to iCC (*p* <0.001) and pCC (*p* <0.05).

### Correlation analyses

Intriguingly, Beclin1 was found to correlate with LC3 in iCC (p<0.05) and eCC (pCC + dCC, p <0.05), also in dCC (p <0.05), and with TOMM20 in iCC (p<0.05); whereas Beclin1 correlated negatively with p62 in dCC (p <0.05) (Table 3). As we observed, LC3 correlated with TOMM20 in eCC (p <0.05), also in pCC (p <0.05), and p62 correlated with TOMM20 in pCC (p <0.05). In addition, Ki-67 was found to correlate negatively with LC3 in eCC (p <0.05), also in dCC (p <0.05), and positively with p62 in iCC, dCC (p <0.05), and with COX4 in iCC (p <0.05). In addition, TOMM20 correlated with COX4 (p <0.05) in HCC.

**Table 3:** The correlations between the levels (immunohistochemistry scores) of investigated proteins in the tumor tissues.

iCC	eCC	pCC	dCC	HCC
Beclin1-LC3 *r = 0*.*60*	Beclin1- LC3 *r = 0*.*60*		Beclin1- LC3 *r = 0*.*61*	
Beclin1-TOMM20 *r = 0*.*41*			Beclin1-p62 *r = -0*.*55*	
	LC3-TOMM20 *r = 0*.*39*	LC3- TOMM20 *r = 0*.*51*		
	LC3-Ki-67 *r = -0*.*36*		LC3-Ki-67 *r = -0*.*42*	
p62-Ki-67 *r = 0*.*42*		p62- TOMM20 *r = 0*.*50*	p62-Ki-67 *r = 0*.*44*	
COX4-Ki-67 *r = 0*.*61*				COX4-TOMM20 *r = 0*.*89*

CC: Cholangiocarcinoma, iCC: Intrahepatic cholangiocarcinoma, eCC: Extrahepatic cholangiocarcinoma, pCC: Perihilar cholangiocarcinoma, dCC: Distal cholangiocarcinoma, HCC: Hepatocellular carcinoma.

The analysis further revealed that LC3 expression was associated with grade (p<0.05, r = 0.51) in pCC. Other correlations were not observed between the levels of investigated proteins and clinicopathologic data neither in CCs nor in HCC.

### Association of immunhistochemical stainings with survival

**Fig 4:**
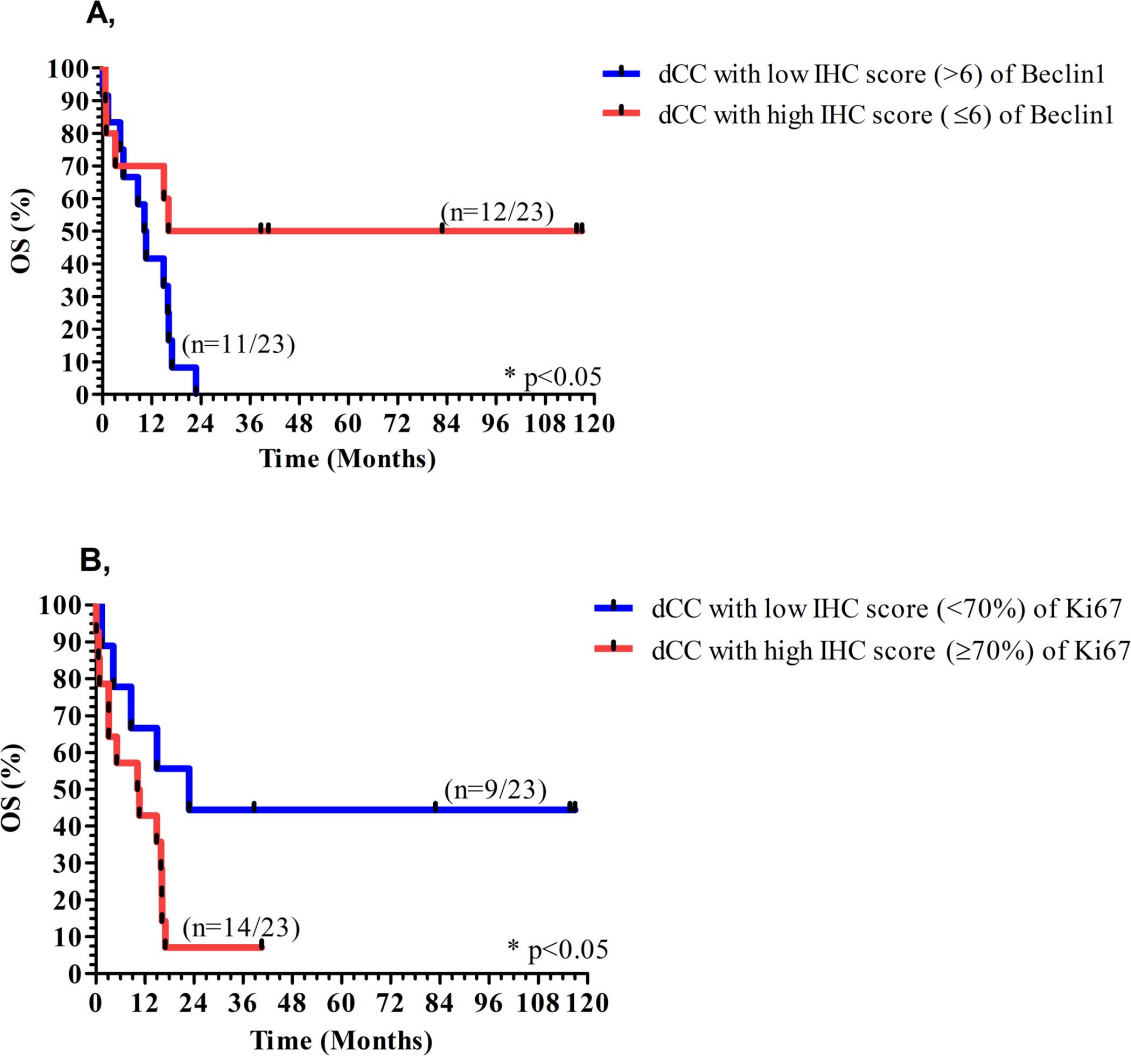
Association of Beclin1 and Ki-67 expression with overall survival in distal cholangiocarcinoma (dCC). Higher level of Beclin1 (A) and lower level of Ki-67 (B) were associated with longer overall survival (OS) in dCC.

In the course of the study, 61 out of the 70 (87.1%) CC patients died; including 24 (out of 28) in the iCC, 19 (out of 19) in the pCC and 18 (out of 23) in the dCC group of patients. The association between protein expression and OS revealed that patients with higher Beclin1 expression in dCC were associated with longer median survival time compared to patients with lower levels of Beclin1 (66.5 versus 10.4 months, p<0.05), as shown in Fig 4A. Further, patients showing lower levels of Ki-67 in dCC were associated with longer median survival time in comparison to patients with higher Ki-67 expression (22.8 versus 10.2 months, p<0.05) (Fig 4B).

### Mitochondrial morphology

**Fig 5:**
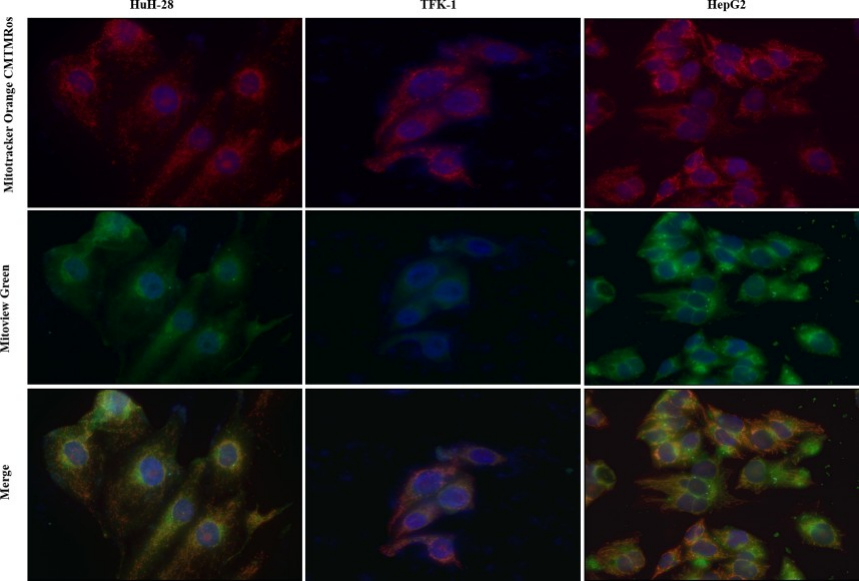
Visualization of mitochondrial morphology in cholangiocarcinoma (CC) and hepatocellular carcinoma (HCC) cell lines. The mitochondria in intrahepatic CC (HuH-28), extrahepatic CC (TFK-1) and HCC (HepG2) cell lines were stained by fluorescent Mitotracker Orange CMTMRos and Mitoview Green dyes (magnification 400x). The former signifies active mitochondria, the latter corresponds to mitochondrial mass.

The morphology of mitochondria was visualized by using Mitotracker Orange CMTMRos and Mitoview Green dyes. The former is a membrane potential-dependent stain, which accumulates in living cells only, reflecting thus the active mitochondria. The latter dye is independent of the membrane potential, providing therefore a read-out related purely to the mitochondrial mass. Under a fluorescent microscope, different reddish and green color patterns were detected in CC and HCC cell lines (Fig 5). The reddish color showed tubular distribution in HepG2 and appeared as a network of mitochondria; however, a clear network could not be observed in HuH-28 and TFK-1 cells, rather it appeared to be disintegrated, revealing much shorter chains of mitochondria and some of them seemed to be punctiform. The green signals were blurred and veiled with a more intensive staining in HuH-28 as compared to TFK-1, whereas the green color revealed rather tubular distribution of mitochondria in HepG2. The merged images disclosed a high percent of orange color in TFK-1, being indicative of the active mitochondria, whereas a considerable amount of green color was recognized in HuH-28 and, in a less degree, in HepG2, as well.

### Visualization of autophagosomes

**Fig 6:**
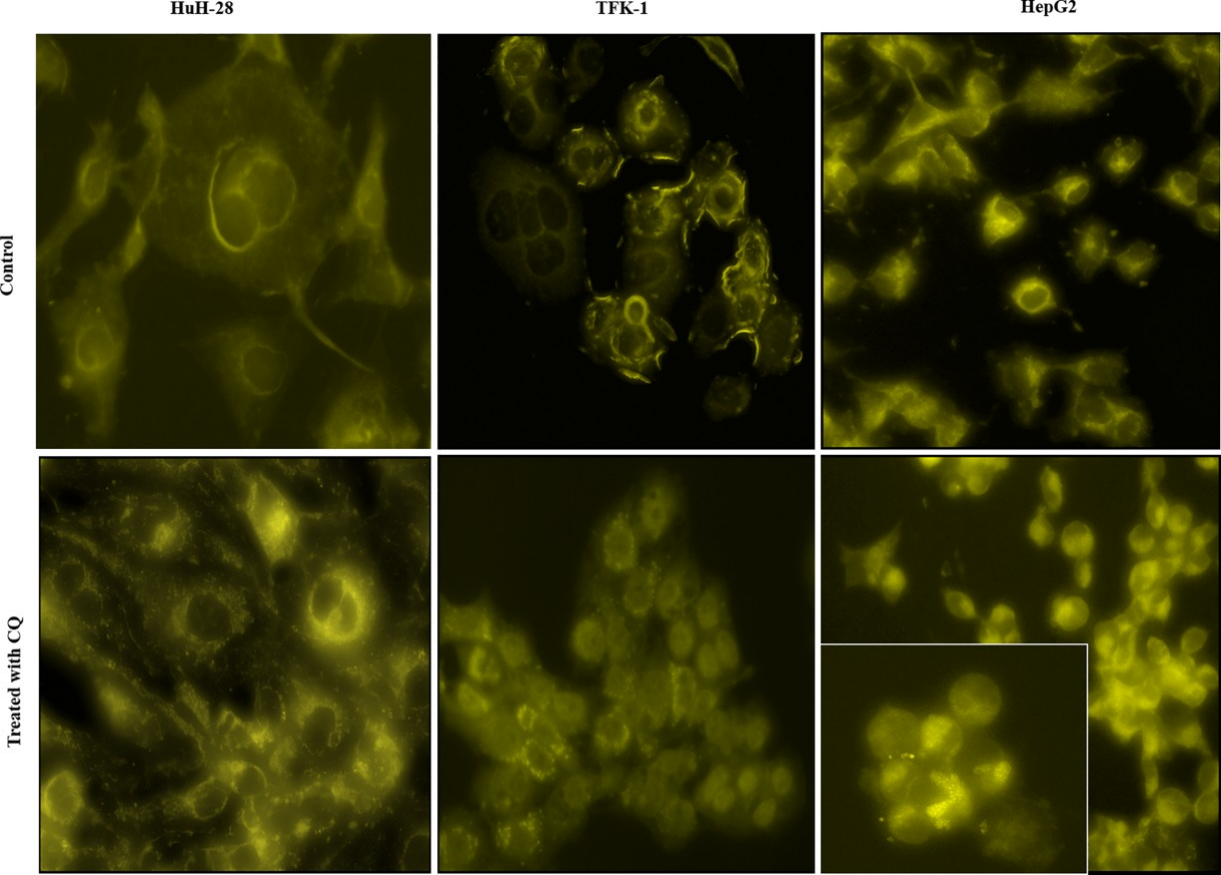
Visualization of autophagosomes in cholangiocarcinoma (CC) and hepatocellular carcinoma (HCC) cell lines. The autophagosomes in intrahepatic CC (HuH-28), extrahepatic CC (TFK-1) and HCC (HepG2) cell lines were stained by Monodansyl-cadaverine (MDC) at states of basal and autophagy inhibition, which was carried out by administering 50 μM of Chloroquine (CQ) for 24 hours prior to detection (magnification 400x, embedded image 600x).

The autophagic vacuoles were visualized by the autofluorescent MDC dye. As shown in Fig 6, diffuse distribution was observed in HuH-28 and TFK-1; however, a punctate pattern appeared in these cell lines upon pretreatment with CQ. Beside the diffuse pattern, HepG2 exhibited a few dot-like signs, and the number of dots seemed to increase slightly following CQ treatment.

### Autophagy induction by Rapamycin

**Fig 7:**
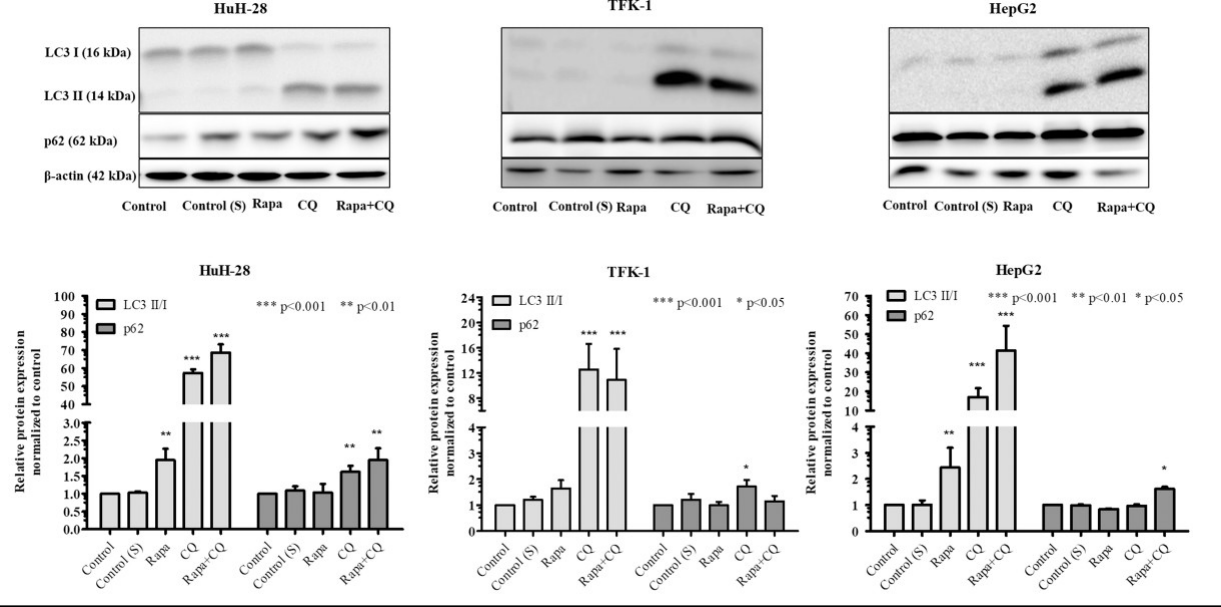
Basal and induced autophagy in cholangiocarcinoma (CC) and hepatocellular carcinoma (HCC) cell lines detected by Western blot. Autophagy was detected by measuring the levels of LC3 I, LC3 II, and p62 in intrahepatic CC (HuH-28), extrahepatic CC (TFK-1) and HCC (HepG2) cell lines at different conditions. Inhibition of autophagy was carried out by administering 50 μM of Chloroquine (CQ) for 24h. Induction of autophagy was performed by administering 0.2 μM of Rapamycin (Rapa) for 24 h. The charts below the images represent the densitometry of LC3 II/I and p62, obtained by taking the (LC3II/β-actin)/(LC3I/β-actin) and p62/β-actin ratios, which were normalized to untreated cells and expressed as mean (n = 3) + S.D. Statistically significant differences are indicated by asterisks (*** *p* <0.001, ** *p* <0.01, * *p* <0.05). S: solvent (DMSO).

To investigate basal autophagy and whether autophagy could be induced in the cell lines, treatments were carried out with CQ, Rapamycin or Rapamycin + CQ. The treatment with CQ in order to inhibit autophagy resulted in increased levels of LC3 II/I in each cell line, whereas p62 was increased in HuH-28 and TFK-1 but not in HepG2 (Fig 7). Rapamycin treatment increased LC3 II/I in HuH-28 and HepG2 without decreased p62; however, Rapamycin + CQ treatment led to increased LC3 II/I in each cell lines and p62 was increased only in HuH-28 and HepG2 cells. Although LC3 II/I was increased in TFK-1 following Rapamycin + CQ treatment, it remained below the level observed following CQ treatment as did p62 as well. Intriguingly, the highest LC3 II/I levels were observed in HuH-28 following CQ and Rapamycin + CQ treatments, and in HepG2 following Rapamycin treatment in comparison to the other two cell lines. The observed increases in the levels of p62 were 1.6-fold in HepG2 following Rapamycin + CQ treatment, 1.6-fold and 1.9-fold in HuH-28 following CQ and Rapamycin + CQ treatment and 1.7-fold in TFK-1 following CQ treatment. Another way of analysis was comparing the unnormalized, β-actin related levels of LC3 II between baseline (CQ–Control) and induced [Rapamycin + CQ]–[Rapamycin]) autophagy states. As revealed, the differences were found to be higher for induced autophagy in HepG2 and HuH-28 (5.7 and 6.5) as compared to baseline (2.4 and 5.5); whereas the difference was higher for baseline state (8.7) compared to induced state (7.4) in TFK-1. Regarding p62, the differences for induced autophagy were higher in HepG2 and HuH-28 (0.98 and 0.4) as compared to the baseline state (0.06 and 0.2); whereas the opposite was found in TFK-1, lower difference for induced state (0.2) and higher difference for baseline (1.16).

### Effect of 5-FU and Sorafenib treatment on autophagy induction

**Fig 8:**
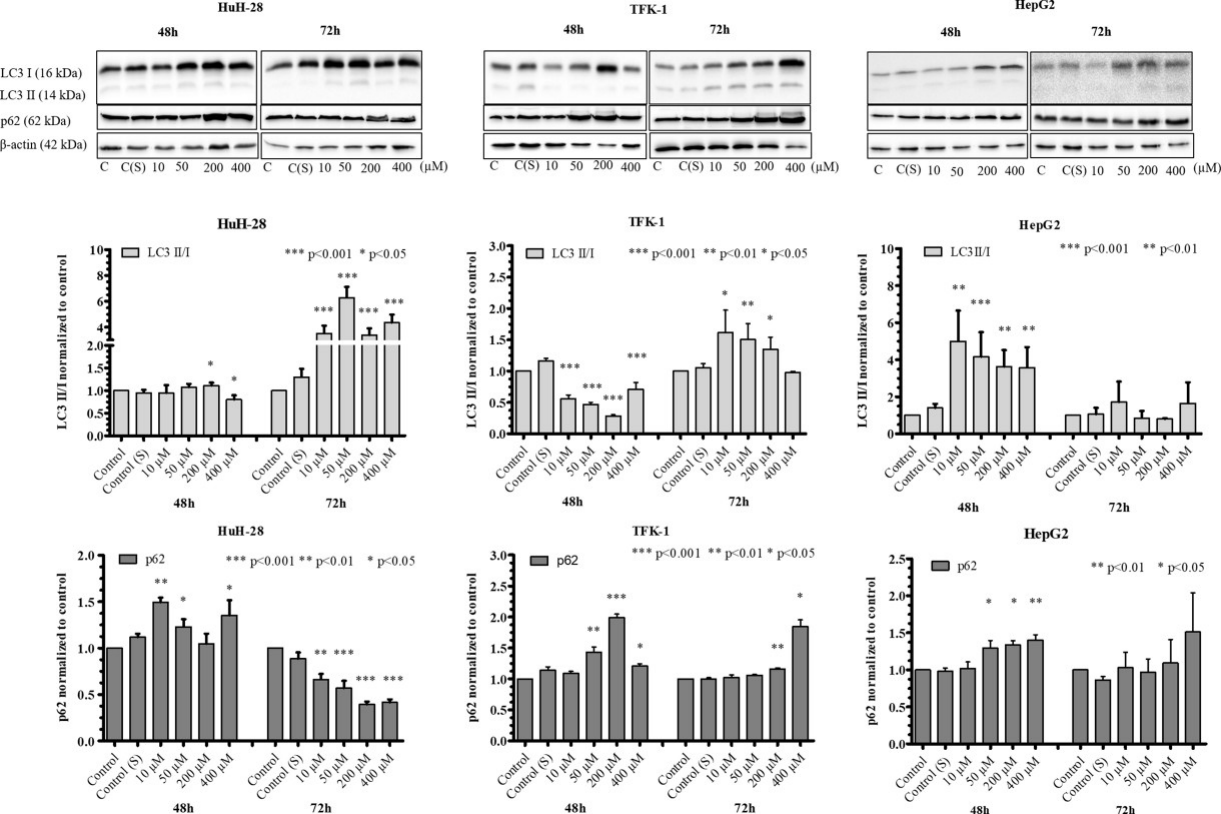
The effect of 5-FU treatment on autophagy induction in cholangiocarcinoma (CC) and hepatocellular carcinoma (HCC) cell lines detected by Western blot. The levels of LC3I, LC3 II, and p62 were detected in intrahepatic CC (HuH-28), extrahepatic CC (TFK-1) and HCC (HepG2) cell lines. Treatments were carried out with administering 10–400 μM of 5-FU for 48 and 72 h. The charts next to the images represent the densitometry of LC3 II/I and p62, obtained by taking the (LC3II/β-actin)/(LC3I/β-actin) and p62/β-actin ratios, which were normalized to untreated cells and expressed as mean (n = 3) + S.D. Statistically significant differences are indicated by asterisks (*** *p* <0.001, ** *p* <0.01, * *p* <0.05). S: solvent (DMSO).

When a chemotherapeutic agent was administered to the cells, the levels of LC3 II/I and p62 varied in association with the cell lines, the applied agent and incubation time. Following 5-FU treatment, increased levels of LC3 II/I were found in HuH-28 at 72 h and in HepG2 at 48 h in comparison to control (Fig 8). The levels of LC3 II/I showed a concentration dependent decrease from 10 to 400 μM in HepG2 at 48 h. Nevertheless, LC3 II/I was slightly increased in TFK-1 at 72 h (10–200 μM), unchanged in HuH-28 at 48 h and in HepG2 at 72 h, and it was lower in TFK-1 at 48 h when compared to control. At the same time, we observed that p62 expression increased in HuH-28 at 48 h but it decreased at 72 h in comparison to control. The levels of p62 were unchanged in HepG2 at 72 h and slightly increased at 48 h (50–400 μM) as compared to control. In TFK-1, the levels of p62 were increased at 48 h (50–400 μM) and at 72 h (200–400 μM) in comparison to control.

**Fig 9:**
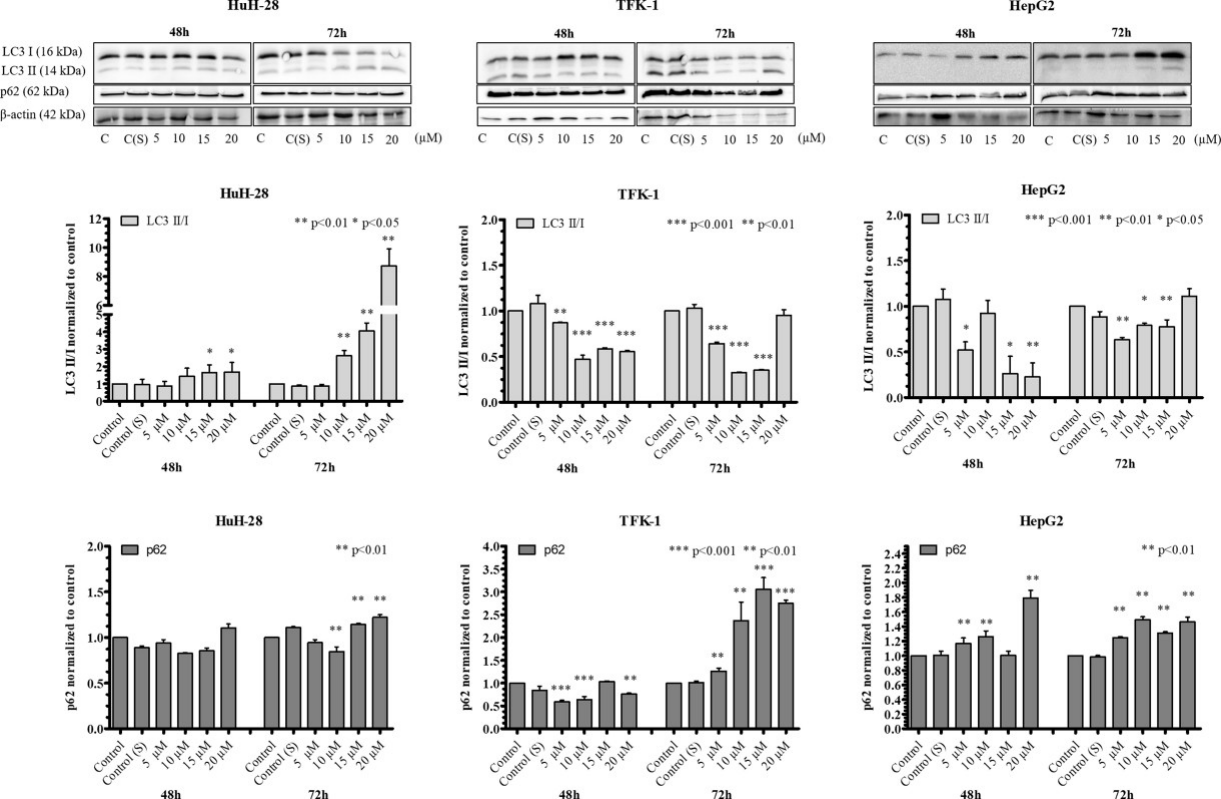
The effect of Sorafenib treatment on autophagy induction cholangiocarcinoma (CC) and hepatocellular carcinoma (HCC) cell lines detected by Western blot. The levels of LC3 I, LC3 II, and p62 were detected in intrahepatic CC (HuH-28), extrahepatic CC (TFK-1) and HCC (HepG2) cell lines. Treatments were performed by administering 5–20 μM of Sorafenib for 48 and 72 h. The charts next to the images represent the densitometry of LC3 II/I and p62, obtained by taking the (LC3II/β-actin)/(LC3I/β-actin) and p62/β-actin ratios, which were normalized to untreated cells and expressed as mean (n = 3) + S.D. Statistically significant differences are indicated by asterisks (*** *p* <0.001, ** *p* <0.01, * *p* <0.05). S: solvent (DMSO).

Following Sorafenib treatment, the levels of LC3 II/I increased dose-dependently in HuH-28 from 10 to 20 μM at 72 h and were also increased at 48 h (15–20 μM) when compared to control cells (Fig 9). In TFK-1, the levels of LC3 II/I were decreased at both 48 (5–20 μM) and 72 h (5–15 μM). In HepG2 cells, the levels of LC3 II/I were decreased at both time points (5, 15, 20 μM at 48 h and 5–15 μM at 72 h) in comparison to control. Decreased levels of p62 were observed in TFK-1 at 5, 10 and 20 μM at 48 h (reaching about 1.5-fold decrease), while the expression of p62 was unchanged in HuH-28 at 48 h. At 72 h, however, the levels of p62 were decreased 10 μM and increased at 15–20 μM (reaching about 1.2-fold increase). The levels of p62 were increased in TFK-1 at 72 h (10–20 μM, more than two-fold) and in HepG2 at both 48 (5, 10 and 20 μM) and 72 h (5–20 μM, not higher than 1.8-fold).

### Effect of 5-FU and Sorafenib treatment on cell viability

**Fig 10:**
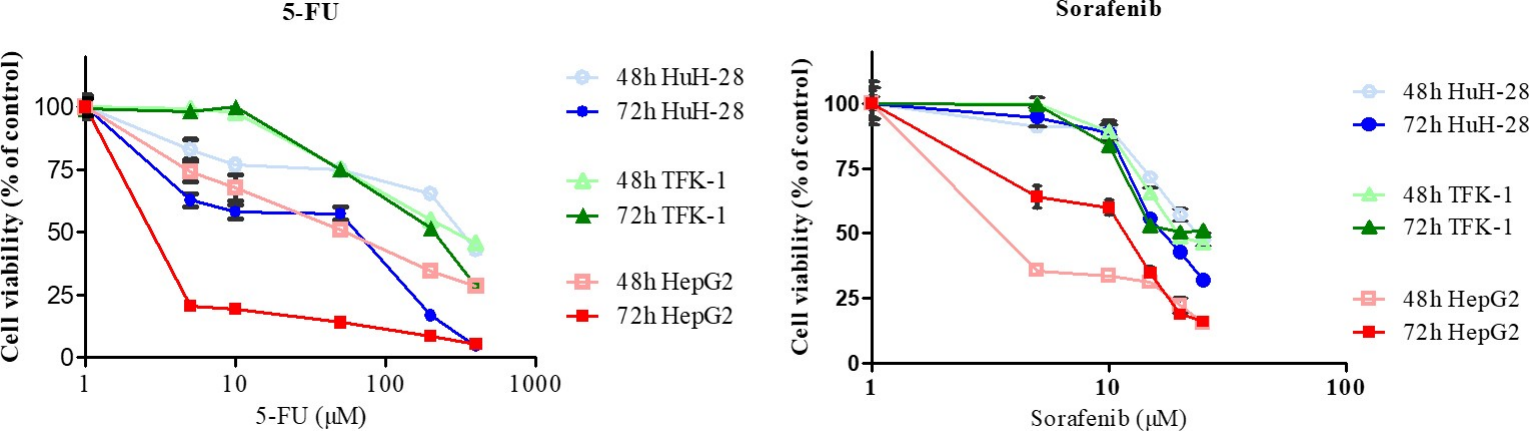
Cytotoxicity of 5-FU and Sorafenib in cholangiocarcinoma (CC) and hepatocellular carcinoma (HCC) cell lines. The intrahepatic CC (HuH-28), extrahepatic CC (TFK-1) and HCC (HepG2) cells were treated with 10–400 μM of 5-FU and 5–25 μM of Sorafenib for 48 and 72 h. Cell viability was determined by the Sulforhodamine B colorimetric assay and expressed as mean (n = 4) ± S.D.

The treatments with increasing concentrations of the chemotherapeutic agents led to decreased cell viability in each cell line (Fig 10). Nevertheless, the cell lines reacted differently. The most prominent reduction of cell viability was observed in HepG2 with both 5-FU and Sorafenib treatment at 48 and 72 h, although Sorafenib at low concentrations led to more reduced viability at 48 h as compared to 72 h. In HuH-28, both 5-FU and Sorafenib resulted in more decreased viability at 72 h, whereas the cell viability percentages were found to be similar at 48 and 72 h following chemotherapy treatments in TFK-1, being these the least reduced percentages observed in the studied cell lines.

We investigated whether inhibition of autophagy would affect cytotoxicity of the cells treated with chemotherapeutic agents As presented in Table 4, both 5-FU and Sorafenib in combination with CQ could reduce the IC50 values in HuH-28 at 48 and 72 h, which was the highest observed reduction in the cell lines. In TFK-1, 5-FU with CQ decreased the IC50 at 48 and 72 h; however, Sorafenib with CQ could not lead to reduced IC50 values. By contrast, HepG2 was found to react with reduced IC50 to 5-FU + CQ treatment at 48 h and to Sorafenib + CQ treatment at 72 h.

**Table 4:** IC50 values in intrahepatic CC (HuH-28), extrahepatic CC (TFK-1) and HCC (HepG2) cells determined following 5-FU and Sorafenib treatment alone or in combination with Chloroquine.

Treatments	IC50 (μM)					
	HuH-28		TFK-1		HepG2	
	48h	72h	48h	72h	48h	72h
**5-FU**	401.4	25.0	285.3	183.4	59.7	3.3
**5-FU + CQ (50 μM)**	3.2	2.9	235.4	152.2	3.0	3.0
**Sorafenib**	24.9	18.7	21.3	21.4	5.6	10.0
**Sorafenib + CQ (50 μM)**	5.0	3.1	25.4	21.0	4.3	2.8

CC: Cholangiocarcinoma, HCC: Hepatocelular carcinoma, CQ: Chloroquine.

## Discussion

Autophagy acts, on the one hand, as a survival mechanism and helps maintain cellular homeostasis under stressful conditions and may hinder oncogenic mutations to occur and tumors to be developed by reducing oxidative stress and DNA damage [12,22]. On the other hand, however, autophagy may be advantageous for established tumors, as it promotes the survival of tumor cells existing in an unbalanced environment lacking oxygen and nutrients [13].

Regarding CC, there are only a few articles investigating the autophagy function in CC lesions and the published data strongly suggest that autophagy is deregulated during cholangiocarcinogenesis, allowing oncogenic transformation [16,17]. CC is a highly heterogeneous tumor in terms of genetics, clinical presentation and progression, and the dominating cisplatin-based therapy may not improve the disease burden significantly. One of the reasons could be that cisplatin-based chemotherapy applied in CC treatment has been reported to induce autophagy, while an inhibited autophagy senzitizes these cells to cisplatin [23].

Since autophagy is involved in CC development and progression and iCC, pCC and dCC have different characteristics, we aimed to reveal differences in the autophagic activity of CC tissues originated from different anatomical locations and cell lines having intrahepatic and extrahepatic origin.

Autophagy is mainly investigated by means of indirect markers. LC3 *per se* indicates that autophagy is initiated but may not reflect upon whether autophagy has proceeded or become impaired [24,25]. A proceeding autophagy implies a low level of p62 since this protein becomes degraded together with the cargo in the autophago-lysosome [26,27]. Beclin1 as an autophagy marker is involved in the initiation of autophagosome formation [13] and there are no evidences whether it enters the autophagosome.

In the first part of the present study, we compared the expression of indirect autophagy and mitochondrial volume markers in the resected CC samples. As we observed in iCC, the expression of LC3 and p62 was increased as compared to non-tumorous tissues and the level of p62 was increased in comparison to eCC. These indicated the presence of an inhibited autophagy in iCC, with occuring autophagosome formation but without considerable autophago-lysosomal degradation. As a support, Sasaki et al. report increased expression of LC3 and p62 by IHC in biliary intraepithelial neoplasia (BilIN-1/2 and BilIN-3) and invasive CC in comparison to normal bile ducts, suggesting that an incomplete autophagy is related to the occurrence and development of CC [16]. In eCC (pCC and dCC), however, we found increased LC3 in comparison to adjacent non-tumorous tissues and decreased p62 as compared to iCC, indicating the presence of a more active autophagy in eCC, with autophagosome formation and autophago-lysosomal degradation.

In pCC, we found increased p62 as compared to non-tumorous surrounding tissues, indicating the presence of an inhibited autophagy, similar to iCC, and p62 was decreased in dCC as compared to iCC, suggesting an active autophago-lysosomal degradation. Further hints for a seemingly inhibited autophagy in iCC and pCC was provided by the similarly high levels of LC3 and p62 observed in HCC as Chava et al. reported elevated p62 in 84% of HCC samples detected by IHC, which could be attributed to impaired autophagic function [28].

The seemingly varying expression of LC3 between the CC groups was not statistically different but the high levels indicate autophagosome formation in CC tissues. This was supported by the finding that Beclin1 correlated with LC3 in iCC and eCC, also in dCC. A more active autopagy in dCC is corroborated by the found negative correlation between Beclin1 and p62.

Decreased Beclin1 expression was observed in dCC as compared to pCC and in HCC as compared to iCC and pCC, indicating a less induced autophagy in both dCC and HCC. Intriguingly, others detected both decreased and increased levels of Beclin1 in other solid tumors [23,29,30], including HCC, in which decreased Beclin1 correlated with HCC grade [31] and low Beclin1 was associated with HCC recurrence [22]. On the other hand, the expression of Beclin1 has been found to be high in chronic hepatitis [30], 76% of iCCs [23], 32% of iCCs and 27% of eCCs (IHC score >6) [29]. All of these data indicate that the level of Beclin1 varies in diseased tissues, including liver. Our anticipation that the level of Beclin1 may not correlate directly with the induction of autophagy is supplied by the fact that Beclin1 is a known tumor suppressor [31]; in particular, it is an inhibitor of cell proliferation, and inactivation of Beclin1 leads to increased tumorigenesis in mice [32,33]. Thus, the decreased Beclin1 level in dCC may be associated with higher tumorigenicity, indicated by the observed higher proliferation rate manifested in high Ki-67 index. Another explanation is that low Beclin1 may also occur when autophagy is induced non-canonically, in a Beclin1-independent manner [34].

In spite of all these, Beclin1 has been reported to be a prognostic factor in iCC and eCC [29,35], and also in other cancers [32,33], as low Beclin1 expression predicts an unfavorable OS and progression-free survival. Our findings were in accordance with this as higher expression of Beclin1 was associated with longer median survival in dCC. Additionally, lower levels of Ki-67 was associated with longer median survival in dCC, suggesting also the prognostic value of Ki-67 in dCC. Data partially support this as Ki-67 expression has been found to be associated with disease stage, nevertheless, without any further subclassification of CC samples [36,37].

In our study, high Ki-67 index was observed in dCC (70%, ranging from 20 to 90%), thus, this group was divided into high and low Ki-67-expression subgroups, revealing further differencies in autophagy function. We found that low Ki-67-expression was associated with increased LC3, Beclin1 and decreased p62 levels as compared to dCC cases revealing high Ki-67 expression and to the non-tumorous surrounding area. This indicated that low proliferating dCCs showed a more active autophagy function with autophagosome formation and considerable autophago-lysosomal degradation, while dCC cases with higher proliferation rate were associated with a less active autophagy function and lower rate for autophagosome formation and autophago-lysosomal degradation. When comparing these two dCC subgroups with pCC and iCC, the lower level of Beclin1 (IHC 5 versus 7) along with high Ki-67-expression in dCC (a Ki-67 index of >70% versus <25%) further seems to support an inverse relation between Beclin1 expression and proliferation.

Using COX4 and TOMM20, we also investigated mitochondrial function in relation to autophagy. COX4 is a mitochondrial respiratory chain protein being essential for ATP production [38], and thus, indicating active mitochondria. TOMM20 is a mitochondrial outer membrane protein involved in protein transport [39], which may be indicative of the mitochondria available in a cell (mitochondrial mass), regardless of being active or damaged. In dCC, we found decreased COX4 expression in the high proliferation group as compared to the low proliferation group. It is known that highly proliferating cells may switch from mitochondrial ATP production to glycolysis in order to maintain the high energy supply [40–42] and liver tumor cells are capable of enhancing glycolysis by inhibiting autophagy in order to supply the energy requirements due to proliferation [43]. Thus, our results seem to indicate this metabolic switch. In pCC, the COX4 expression was not different from the other CC subtypes, whereas TOMM20 was found to correlate with both LC3 and p62, suggesting mitophagy in pCC [27,44]. In HCC, on the contrary, the found correlation of COX4 with TOMM20 indicated that mitochondria in HCC were likely to be active. As a support, it has been observed that oxidative phosphorylation is less impaired in a partially differentiated HCC cell line compared to an undifferentiated HCC cell line [45]. This was indicated by our data as well, as 66.7% of our HCC cases were found to be moderately differentiated. Additionally, a correlation was further observed between the levels of LC3 and the degree of differentiation in pCC, suggesting an increased autophagosome formation in high grade pCCs. To support this, more autophagic vacuoles have been detected in tubular adenocarcinomas with higher grade [46].

In the second part of the present study, we examined basal and induced autophagy in CC and HCC cell lines. Basal autophagy was investigated by examining mitochondrial morphology, the presence of autophagic vacuoles and the levels of LC3 and p62.

Mitotracker Orange dye revealed fragmented mitochondria in iCC (HuH-28) and eCC (TFK-1) cells; however, signs of inactive mitochondria also appeared with Mitoview Green dye in iCC and eCC but much less in the latter. Fragmentation of mitochondria is known to indicate mitochondrial damage [47] and thus, the observed less inactive mitochondria in eCC cells suggests a more effective elimination of defective mitochondria in this cell line. On the contrary, the network of mitochondria with elongated morphology indicates mainly functioning mitochondria in the HCC-derived cell line (HepG2). As a support, the elimination of mitochondria by autophagy is avoided in the presence of mitochondrial elongation and sustained ATP production [44,47]. In addition, the autophagosomes became visible in the iCC and eCC cells when these cells were treated with CQ in advance of MDC staining. Since CQ is a known autophagy inhibitor impeding the fusion of autophagosomes with lysosomes [48], our results indicates the presence of active autophagy in these cell lines [25,49,50]. In HepG2, however, the appearing punctate signs, which could not be increased significantly by CQ treatment, suggests a lower autophagic activity. Analyzing LC3 II/I ratio and the level of p62 as indirect autophagy markers [25,50], significantly increased LC3 II/I ratio was observed in iCC and HepG2 cells and moderate increase in TFK-1 following CQ treatment as compared to the controls. In parallel, the increase of p62 expression was far below in magnitude in comparison to LC3 II/I ratio and was observed in iCC and eCC cell lines (1.5 times increase), with no increase in HepG2 as compared to control. These indicate an active autophagosome formation in the cell lines; however, autophagosome-related lysosomal degradation seemed to occur in iCC and eCC cell lines. Taken together, these data support the presence of basal autophagy in iCC and eCC cells; whereas autophagy seemed to be initiated in HepG2 but our results did not support a p62-related autophago-lysosomal degradation in this cell line.

For induction of autophagy activity, we chose the mTOR pathway inhibitor Rapamycin because it is the major negative regulator of autophagy [51,52], mTOR pathway plays a central role in cell proliferation and growth, and inhibition of mTOR function can be a strategic approach in CC and HCC [13,53]. Further, no Rapamycin-induced autophagy has been reported in CC to the best of our knowledge. Our results revealed that Rapamycin could induce autophagy in iCC and HepG2 cells, indicated by the increased levels of LC3 II/I and p62 following Rapamycin + CQ treatment as compared to Rapamycin alone. In the eCC cells, however, the levels of both LC3 II/I and p62 were found to be lower in comparison to the baseline (CQ treatment). We also compared the levels of LC3 II and p62 between induced ([Rapamycin + CQ]—[Rapamycin]) and baseline (CQ—Control) states [54]. The observed higher values for induced state in iCC and HepG2 cell lines as compared to the baseline further confirmed induction of autophagy, which seemed to be more pronounced in iCC as compared to HepG2; whereas no autophagy induction was indicated by this comparison in eCC cells. Cells have been reported to have different response to Rapamycin, ranging from sensitivity up to complete resistance [55]. According to previous publications, Rapamycin treatment reduces mTOR activity in HepG2 without leading to down-regulated proliferation [56]; however, the Rapamycin derivative Everolimus abolishes mTOR activation in eCC cells with significant inhibition of proliferation [57]. It seems from all these data that induction of autophagy by Rapamycin depends on cellular factors in iCC and eCC cell lines.

Autophagy is not only adapted by tumor cells for survival but it may also enhance resistance to antitumor agents [12]. The dominating cisplatin-based therapy may not lead to remission and its severe side effects limit the use of second-line chemotherapy agents [8]. Therefore, we intended to learn whether autophagy is inducible in the examined CC and HCC cell lines by 5-FU and Sorafenib as second-line chemotherapies and possible treatment options in CC [58,59].

Increased autophagy activity was observed following 5-FU treatment in iCC, indicated by the increased levels of LC3 II/I and decreased expression of p62 in comparison to control, which was more active at a later time point. In HepG2 cell line, the levels of LC3II/I showed a concentration-dependent decrease from low to high concentration of 5-FU with slightly increasing p62 expression in parallel, indicating that autophagy became gradually less active at higher concentration of 5-FU, which activation seemed to disappear with incubation time. As we observed higher IC50 in HepG2 at 48 h when autophagy was induced, it seems that autophagy induction by 5-FU contributed to a higher proliferation rate in HepG2. In iCC cells, however, the lower IC50 at 72 h when autophagy was induced suggests that induction of autophagy occurred at the effect of prolonged 5-FU treatment, which was likely to increase survival of iCC cells. The eCC cells, however, showed decreased autophagy activity at early time point, indicated by the decreased levels of LC3 II/I and increased expression of p62, which seemed to normalize at later time point. The fact that eCC cells were less sensitive to 5-FU treatment was indicated by the higher IC50 values at both time points, and this may explain why 5-FU treatment did not induce autophagy in this cell line. Previously, 5-FU has been demonstrated to induce autophagy in HCC and eCC cell lines [59,60]; nevertheless, the eCC cell line differs from our chosen eCC cell line, suggesting a cell line-dependent autophagy induction response to 5-FU. Our findings with HepG2 cells are in agreement with previous data concerning the increased levels of LC3 II [61], and an IC50 value of 42.57 μM at 48 h following 5-FU treatment, although the authors report concentration-dependently increased LC3 II and decreased p62 levels [62].

Sorafenib treatment increased the autophagy activity of iCC cell line, indicated by the slightly increased LC3 II/I at early time point and the dose-dependent increase of LC3 II/I at later time point, which was also characterized by slightly increased p62 expression. On the contrary, HepG2 cells showed decreasing LC3 II/I and increasing p62 expression and eCC cells decreasing LC3 II/I with decreasing p62 and gradually decreasing LC3 II/I and increasing p62 expression, indicating decreased autophagy activity in these cell lines following Sorafenib treatment. Previously, Sorafenib has been found to induce autophagy in HCC using therapeutic dose (10 μM); however, this induction was transiently active and faded away within a day following treatment [63,64]. It is known that diverse intracellular molecular interactions are able to alter autophagy induction in response to Sorafenib [65–67], which may explain the different autophagic response of iCC, eCC and HCC cell lines to Sorafenib. Additionally, Sorafenib has been found to inhibit cell growth less effectively in CC cells compared to HCC cells [68], which is supported by our observed IC50 values. Taken together, 5-FU and Sorafenib seemed to induce autophagy in iCC, and 5-FU in HepG2 cell lines. However, our results did not support autophagy induction in eCC cells following 5-FU and Sorafenib treatments.

Investigating the effect of chemotherapy treatment on proliferation in combination with autophagy inhibitor CQ revealed significantly decreased IC50 values in iCC cells with both 5-FU and Sorafenib, and in HepG2 cells with 5-FU at early time point. This is in line with our autophagy induction data observed following 5-FU and Sorafenib treatments, suggesting that CQ sensitized iCC cells to 5-FU and Sorafenib, and HepG2 cells to 5-FU by inhibiting not only induced but basal autophagic activity as administration of CQ decreased cell viability by 71% in iCC and 81% in HepG2 cells, and by 6.5% in eCC cells (S2 Fig). This indicates that basal autophagy is an important component for the survival of iCC and HepG2 cells. Autophagy, however, seems to play a less important role in the life of eCC cells, which was also supported by the only slightly reduced IC50 values observed in these cells following 5-FU + CQ treatment.

In summary, our study revealed differences in the autophagy activities of CC tissues and cells originated from different anatomical locations. Regarding the resected tumor tissues, the indirect markers indicated an inhibited autophagy in iCC, while eCC showed mixed autophagy characteristics: pCC resembled iCC, whereas dCC displayed an active autophagy, which occurred in association with low Ki-67 index. In dCC, low levels of Beclin1 and high levels of Ki-67 were associated with poor OS, suggesting the prognostic role of these proteins in dCC. The in vitro studies revealed the presence of basal autophagy in each cell lines and Rapamycin was able to induce autophagy in iCC and HepG2 cells. Second-line chemotherapy agents seemed to induce autophagy in a cell line-dependent manner: 5-FU induced autophagy in iCC and HepG2 cells, and Sorafenib did so in iCC. The chemotherapy agents when administered in combination with autophagy inhibitor CQ seemed to decrease IC50 effectively in those cell lines where basal and/or induced autophagy were present, suggesting that the use of autophagy inhibitor along with chemotherapy for the treatment of CC may be beneficial to reduce cell proliferation.

## Supporting information

S1 Fig Expression of COX4 in cholangiocarcinoma (CC) subtypes and hepatocellular carcinoma (HCC) detected by immunohistochemistry (IHC). No statistical difference was found between the IHC scores for CC subtypes and HCC. The red thick horizontal lines represent the median. Statistical analysis was carried out by using Kruskal-Wallis analysis of variance. iCC: intrahepatic CC, eCC: extrahepatic CC, pCC: perihilar CC, dCC: distal CC.
(DOCX)

S2 Fig Cytotoxicity of Chloroquine (CQ) in cholangiocarcinoma (CC) and hepatocellular carcinoma (HCC) cell lines. The intrahepatic CC (HuH-28), extrahepatic CC (TFK-1) and HCC (HepG2) cells were treated with 50 μM of CQ for 48 and 72 h. Cell viability was determined by the Sulforhodamine B colorimetric assay and expressed as mean (n = 3) ± S.D. of the absorbance ratio normalized to untreated cells.
(DOCX)

S1 Raw images (PDF)
