# Supplementary material for: Autophagy activity in cholangiocarcinoma is associated with anatomical localization of the tumor
Source: PLoS One. 2021 Jun 15;16(6):e0253065. doi: 10.1371/journal.pone.0253065 (PMC8205141; doi:10.1371/journal.pone.0253065)
Supplement: S1 Fig — No statistical difference was found between the IHC scores for CC subtypes and HCC. The red thick horizontal lines represent the median. Statistical analysis was carried out by using Kruskal-Wallis analysis of variance. iCC: intrahepatic CC, eCC: extrahepatic CC, pCC: perihilar CC, dCC: distal CC. (DOCX) [file pone.0253065.s001.docx]

**S1 Fig. Expression of COX4 in cholangiocarcinoma (CC) subtypes and hepatocellular carcinoma (HCC) detected by immunohistochemistry (IHC).** No statistical difference was found between the IHC scores for CC subtypes and HCC. The red thick horizontal lines represent the median. Statistical analysis was carried out by using Kruskal-Wallis analysis of variance. iCC: intrahepatic CC, eCC: extrahepatic CC, pCC: perihilar CC, dCC: distal CC.
