# Supplementary material for: Autophagy activity in cholangiocarcinoma is associated with anatomical localization of the tumor
Source: PLoS One. 2021 Jun 15;16(6):e0253065. doi: 10.1371/journal.pone.0253065 (PMC8205141; doi:10.1371/journal.pone.0253065)
Supplement: S2 Fig — The intrahepatic CC (HuH-28), extrahepatic CC (TFK-1) and HCC (HepG2) cells were treated with 50 μM of CQ for 48 and 72 h. Cell viability was determined by the Sulforhodamine B colorimetric assay and expressed as mean (n = 3) ± S.D. of the absorbance ratio normalized to untreated cells. (DOCX) [file pone.0253065.s002.docx]

**S2 Fig. Cytotoxicity of Chloroquine (CQ) in cholangiocarcinoma (CC) and hepatocellular carcinoma (HCC) cell lines.** The intrahepatic CC (HuH-28), extrahepatic CC (TFK-1) and HCC (HepG2) cells were treated with 50 μM of CQ for 48 and 72 h. Cell viability was determined by the Sulforhodamine B colorimetric assay and expressed as mean (n=3) ± S.D. of the absorbance ratio normalized to untreated cells.
