# Supplementary material for: Autophagy activity in cholangiocarcinoma is associated with anatomical localization of the tumor
Source: PLoS One. 2021 Jun 15;16(6):e0253065. doi: 10.1371/journal.pone.0253065 (PMC8205141; doi:10.1371/journal.pone.0253065)

Original gels

Symbols/Abbreviations:

MW: molecular weight

NC: negative control

X: lanes not included in the final figure

C: Control (untreated) cells

C(S): solvent (DMSO) treated cells

Rapa: Rapamycin

CQ: Chloroquine

Original Western blots of Fig 7

The method used to capture the images: *G:BOX* Chemi XR 5 image *documentation system (Syngene, Cambridge, UK).*

The membranes were cut into more pieces based on the MW wells

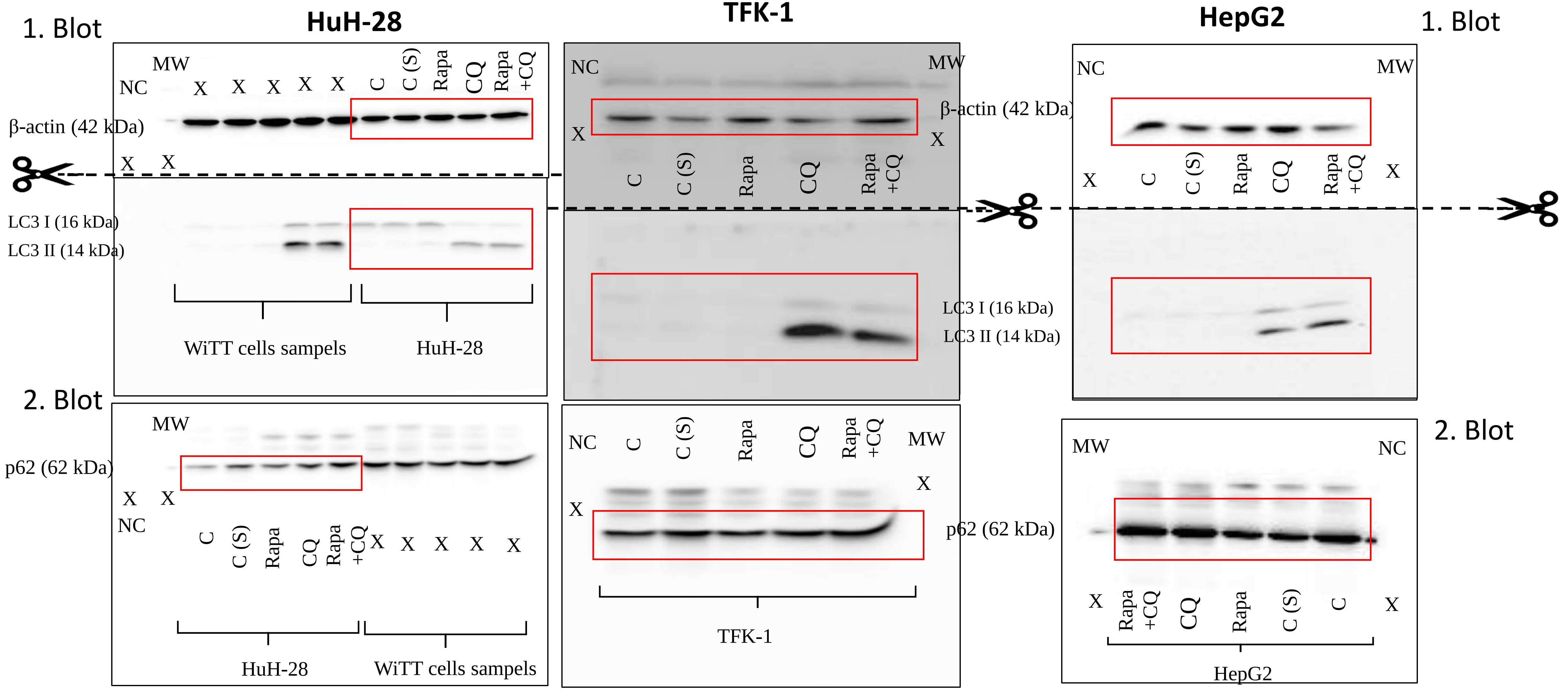

Original Western blots of Fig 8 (HuH-28)

The method used to capture the images: *G:BOX* Chemi XR 5 image *documentation system (Syngene, Cambridge, UK).*

1 Blot

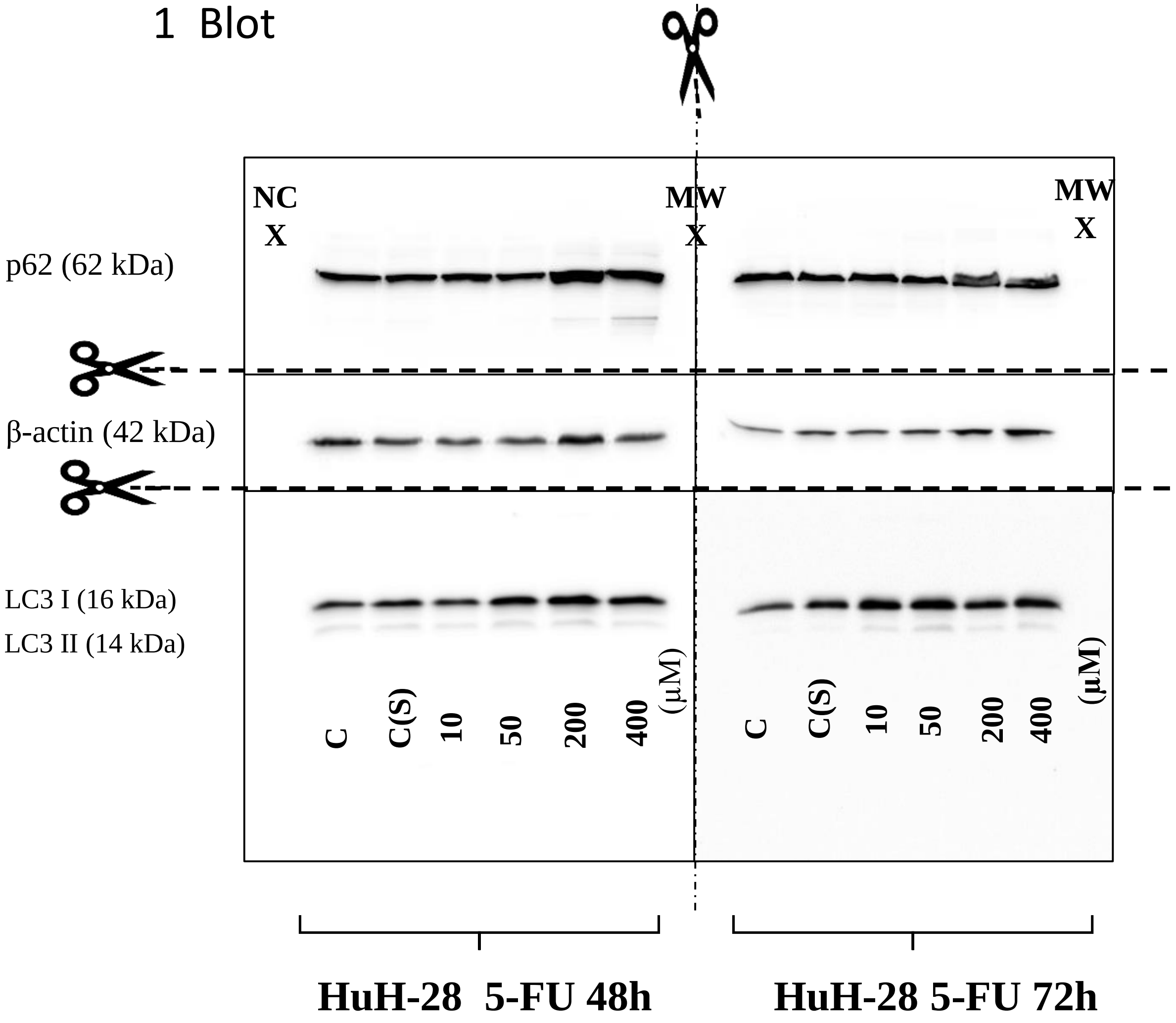

Original Western blots of Fig 8 (TFK1)

The method used to capture the images: *G:BOX* Chemi XR 5 image *documentation system (Syngene, Cambridge, UK).*

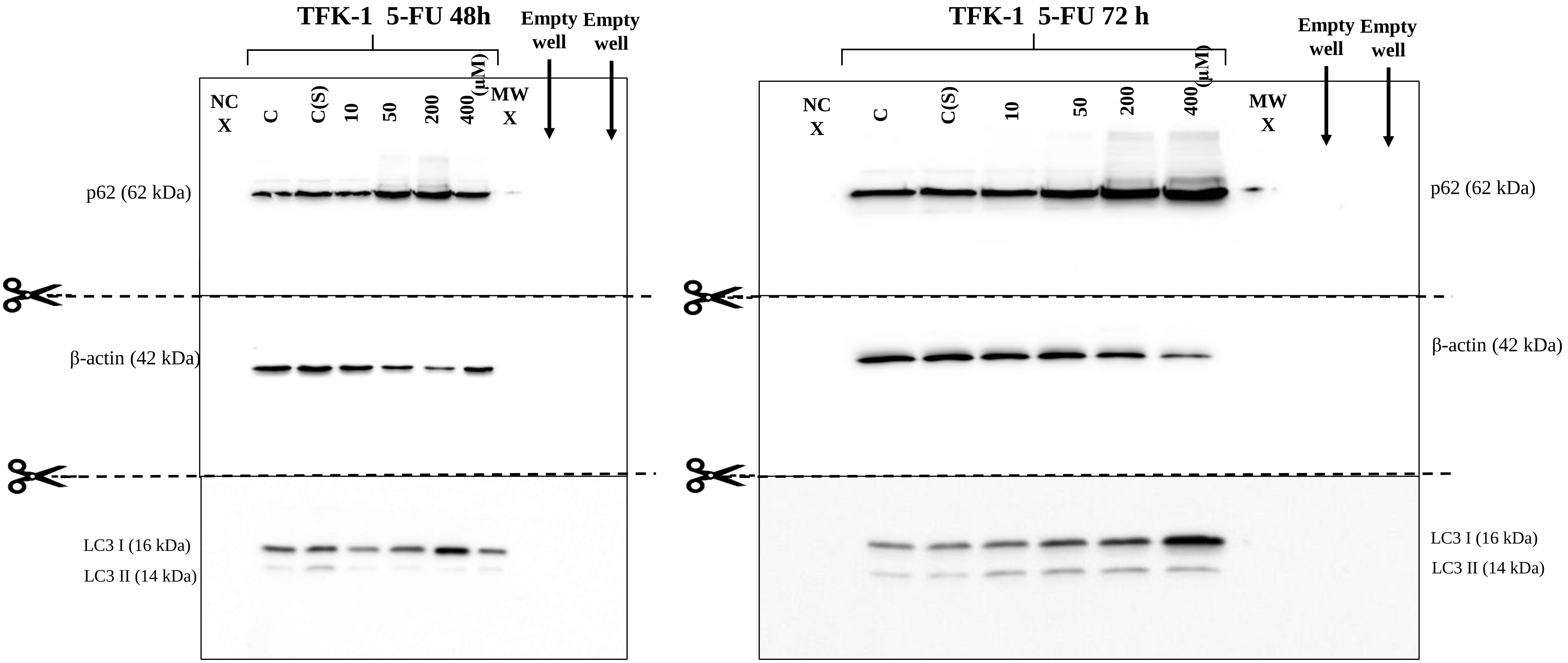

Original Western blots of Fig 8 (HepG2)

The method used to capture the images: *G:BOX* Chemi XR 5 image *documentation system (Syngene, Cambridge, UK).*

1. Blot

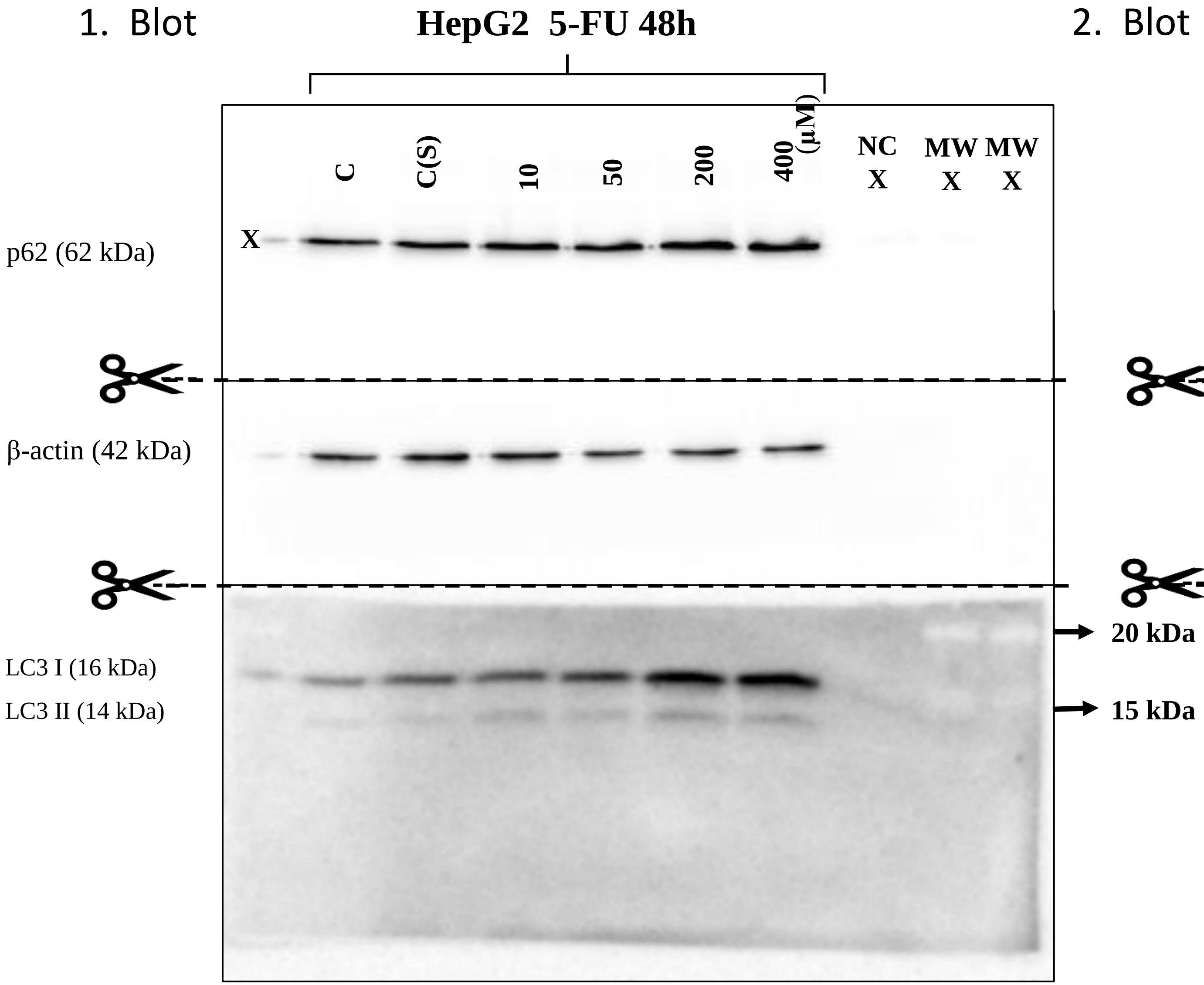

2. Blot

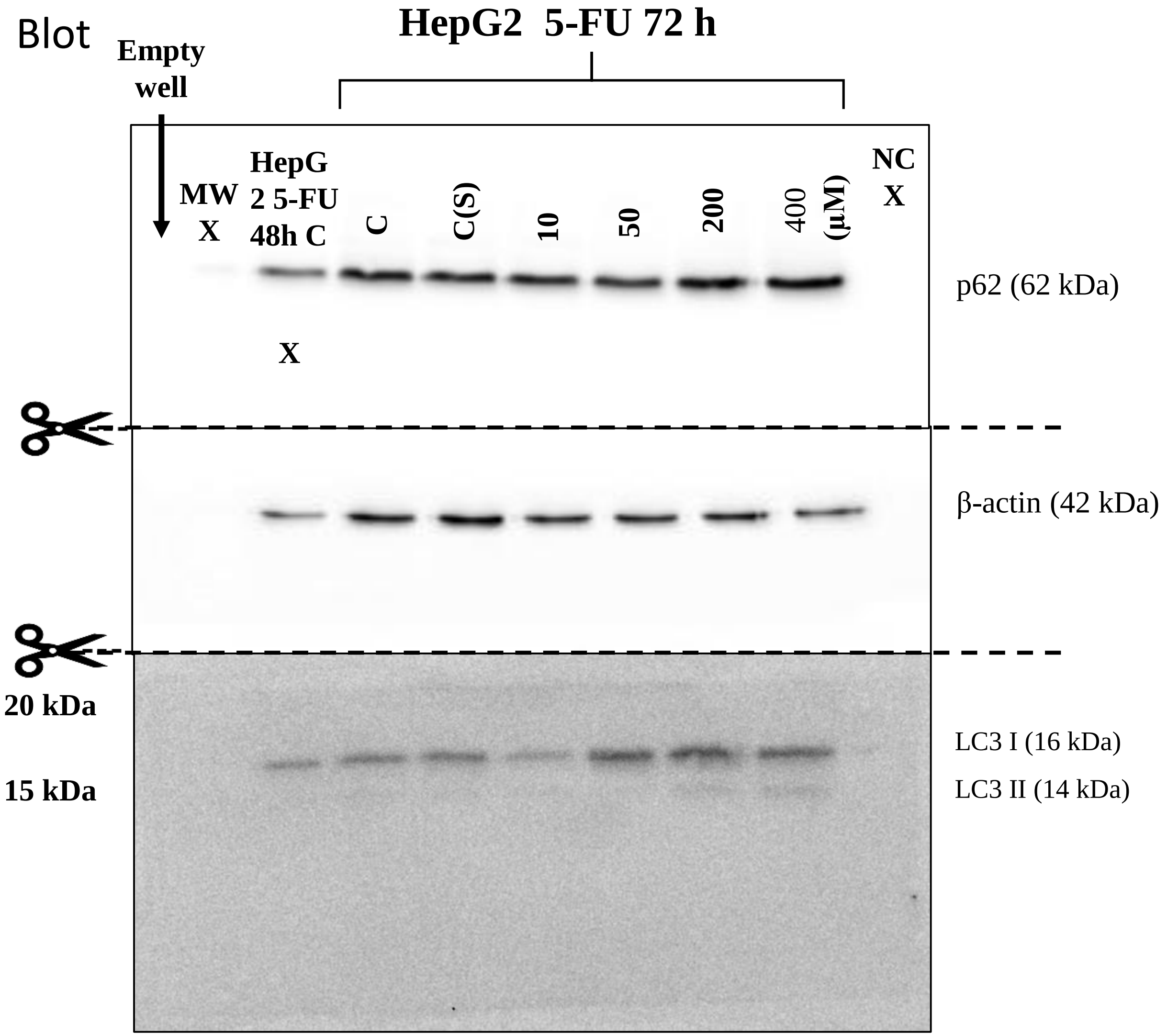

Original Western blots of Fig 9 (HuH-28)

The method used to capture the images: *G:BOX* Chemi XR 5 image *documentation system (Syngene, Cambridge, UK).*

1. Blot

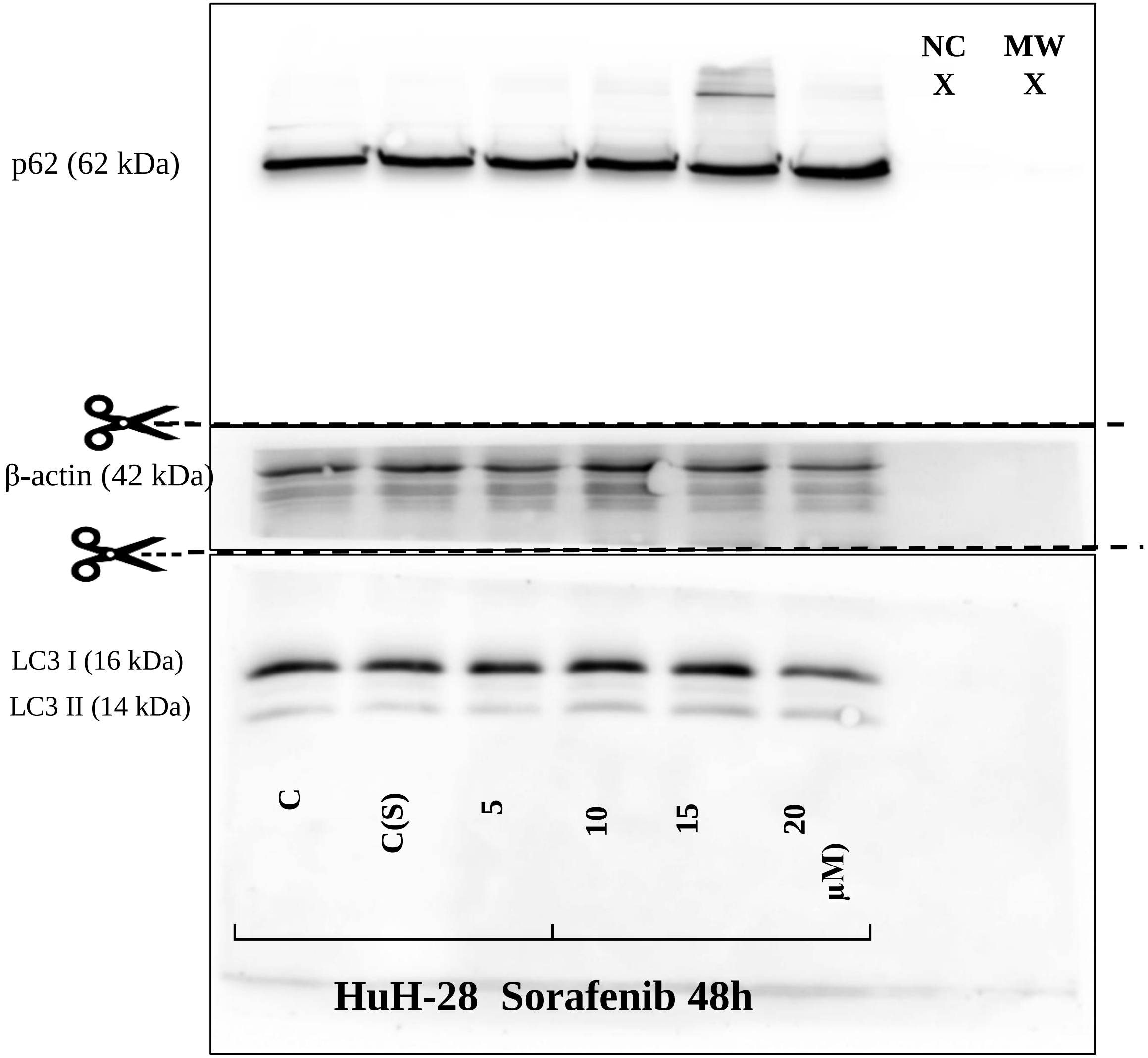

2. Blot

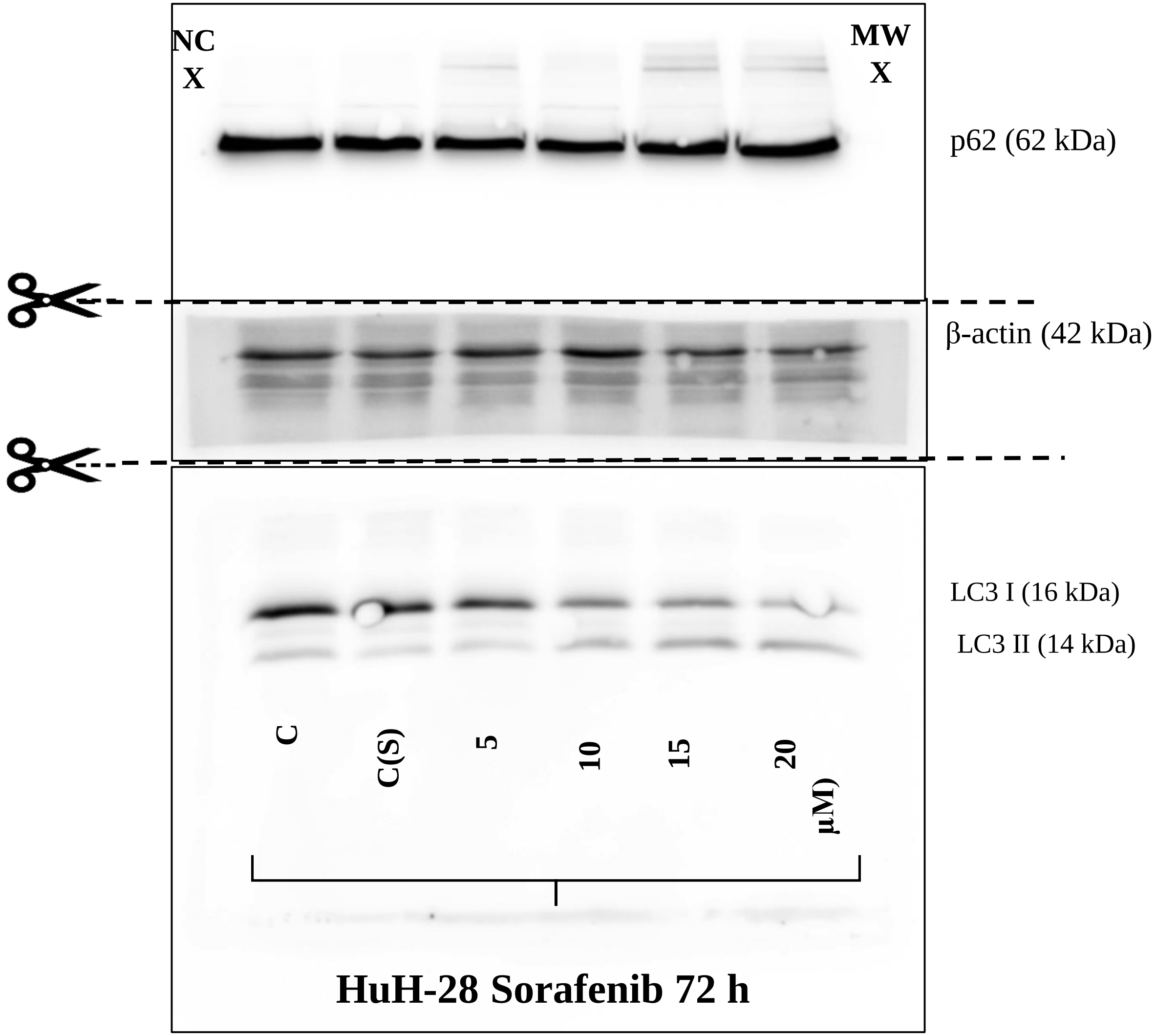

Original Western blots of Fig 9 (TFK-1)

The method used to capture the images: *G:BOX* Chemi XR 5 image *documentation system (Syngene, Cambridge, UK).*

1. Blot

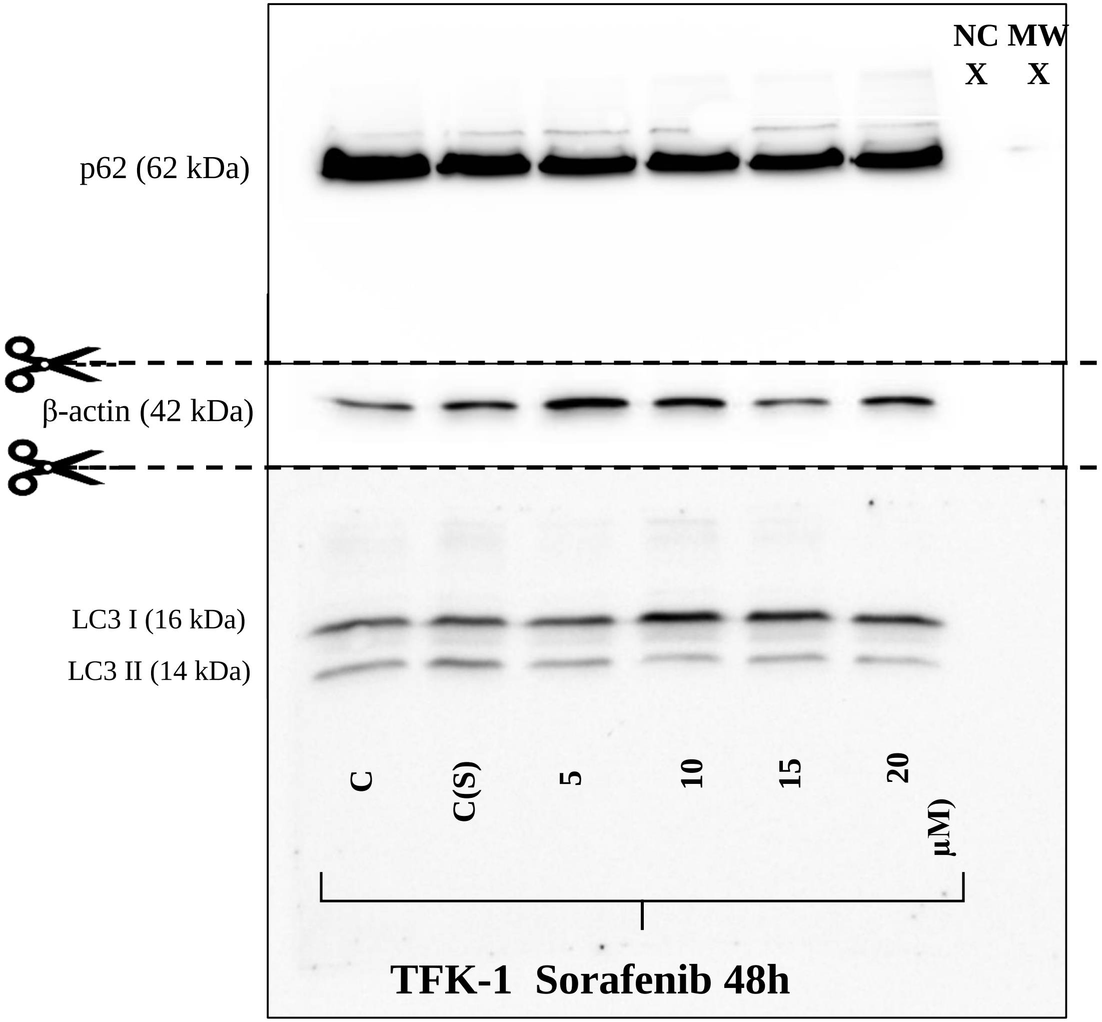

2. Blot

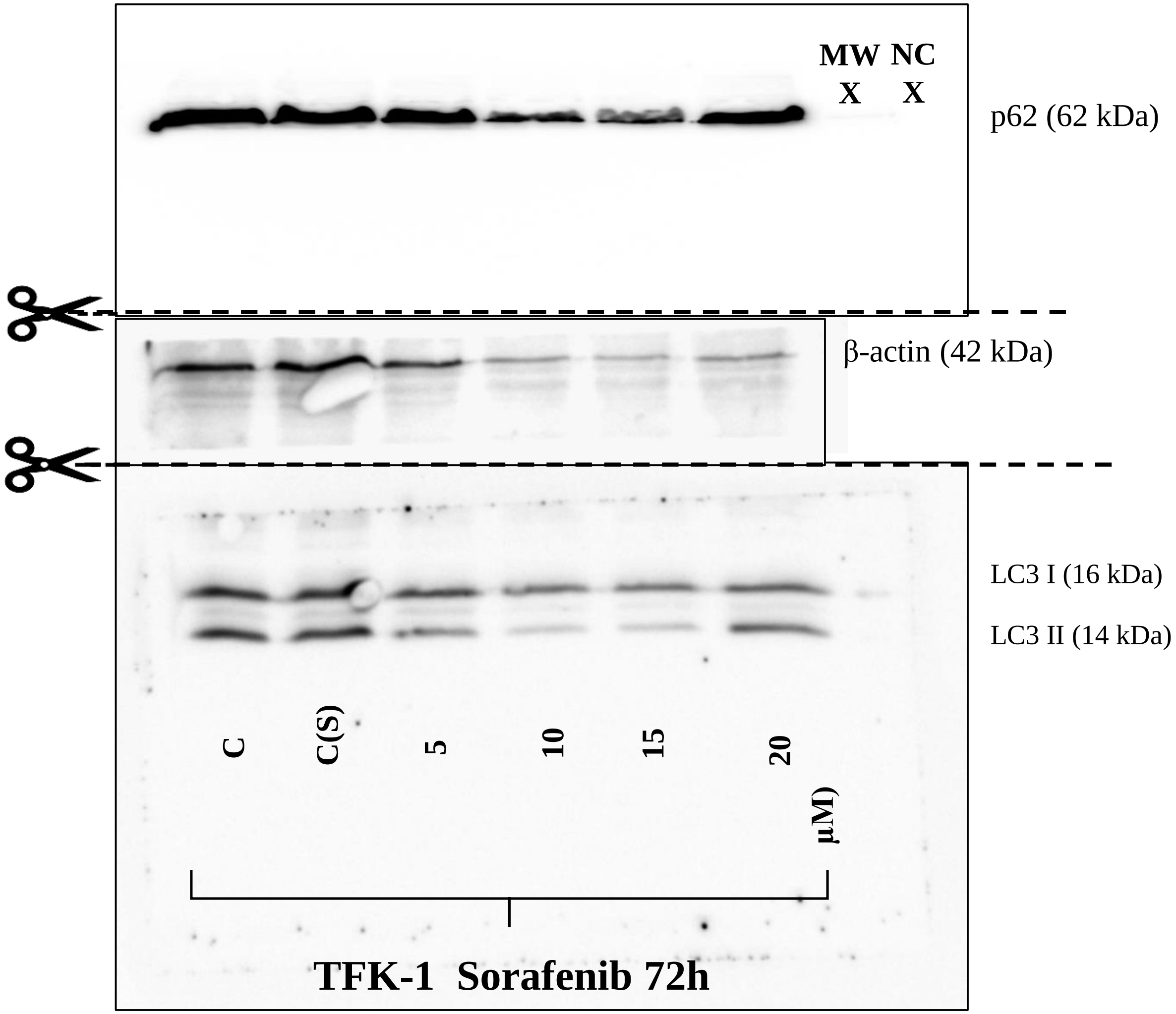

Original Western blots of Fig 9 (HepG2)

The method used to capture the images: *G:BOX* Chemi XR 5 image *documentation system (Syngene, Cambridge, UK).*

1. Blot

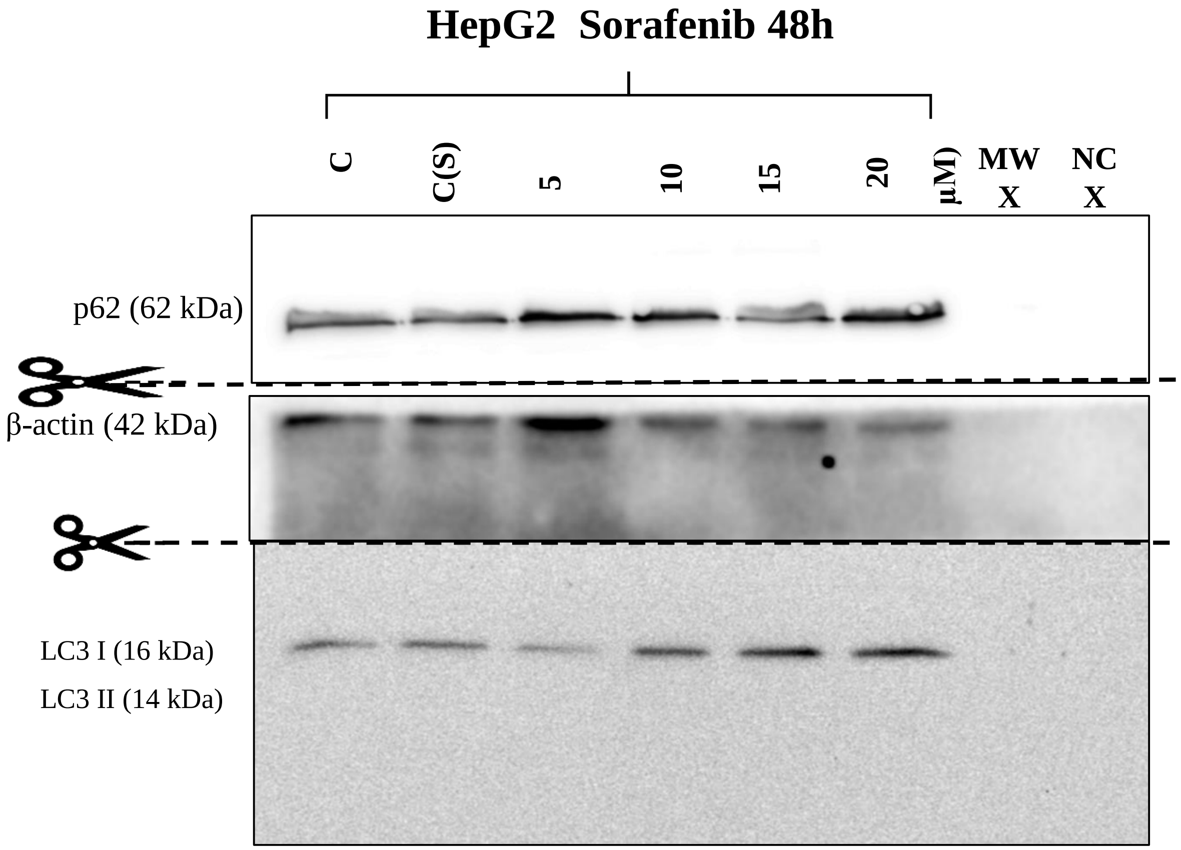

2. Blot

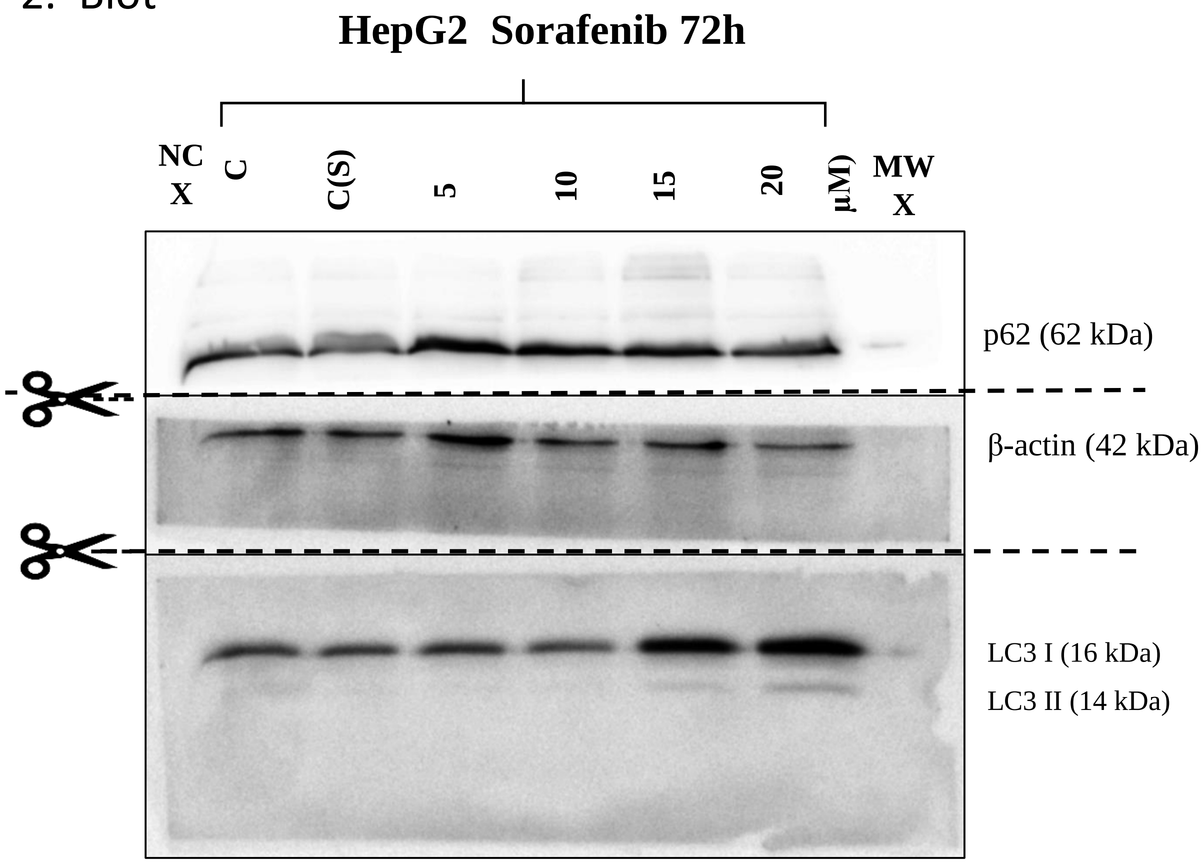

Supplement: S1 Raw images — (PDF) [file pone.0253065.s003.pdf]
